# Highly active earth-abundant ammonia cracking catalysts using long chain hydrocarbon stabilised colloidal nanoparticles

**DOI:** 10.1039/d6cy01170e

**Published:** 2026-10-26

**Authors:** Julien Mahin, Joseph El-Kadi, Laura Torrente-Murciano

**Affiliations:** a Department of Chemical Engineering and Biotechnology, University of Cambridge Philippa Fawcett Drive CB3 0AS Cambridge UK lt416@cam.ac.uk

## Abstract

In this work, we report the synthesis of highly active non-noble metal catalysts with controlled nanoparticle size and dispersion through a new catalyst preparation strategy, combining the continuous-flow synthesis of size-controlled Fe, Co and Ni nanoparticles by thermal decomposition of zero-valent precursors with their direct impregnation *via* the so-called colloidal incipient wetness impregnation (CIWI). The use of long-chain hydrocarbon ligands provides not only size control and colloidal stability at high nanoparticle concentration but also the synergetic simultaneous removal and metal reduction at low temperatures. Indeed, we demonstrate that the removal of these ligands is catalysed by Co and Ni particles, enabling their simultaneous reduction at lower temperatures and thus, avoiding their agglomeration. This combination of controlled nanoparticle synthesis, high-concentration processing and *in situ* self-cleaning provides a route to size control that is difficult to access by conventional incipient wetness impregnation. The flexibility of CIWI further enables systematic investigation of nanoparticle size–activity relationships independently of support effects. Applied to ammonia decomposition, Ni/CeO_2_ exhibits a pronounced size dependence, with 2.4 nm Ni nanoparticles delivering an activity of 3130 mol_NH_3__ mol_metal_^–1^ s^–1^ at 500 °C, ranking among the most active earth-abundant catalysts reported to date and approaching the performance of state-of-the-art Ru-based systems. The CIWI therefore provides a versatile platform for designing highly active, size-controlled non-noble metal catalysts and is broadly applicable to other metal–support combinations and catalytic reactions.

## Abstract 2

Long-chain hydrocarbon ligands enable the size-controlled synthesis of non-noble metal nanoparticles at low reduction temperatures, resulting in highly active NH_3_ cracking catalysts.

## Graphical abstract

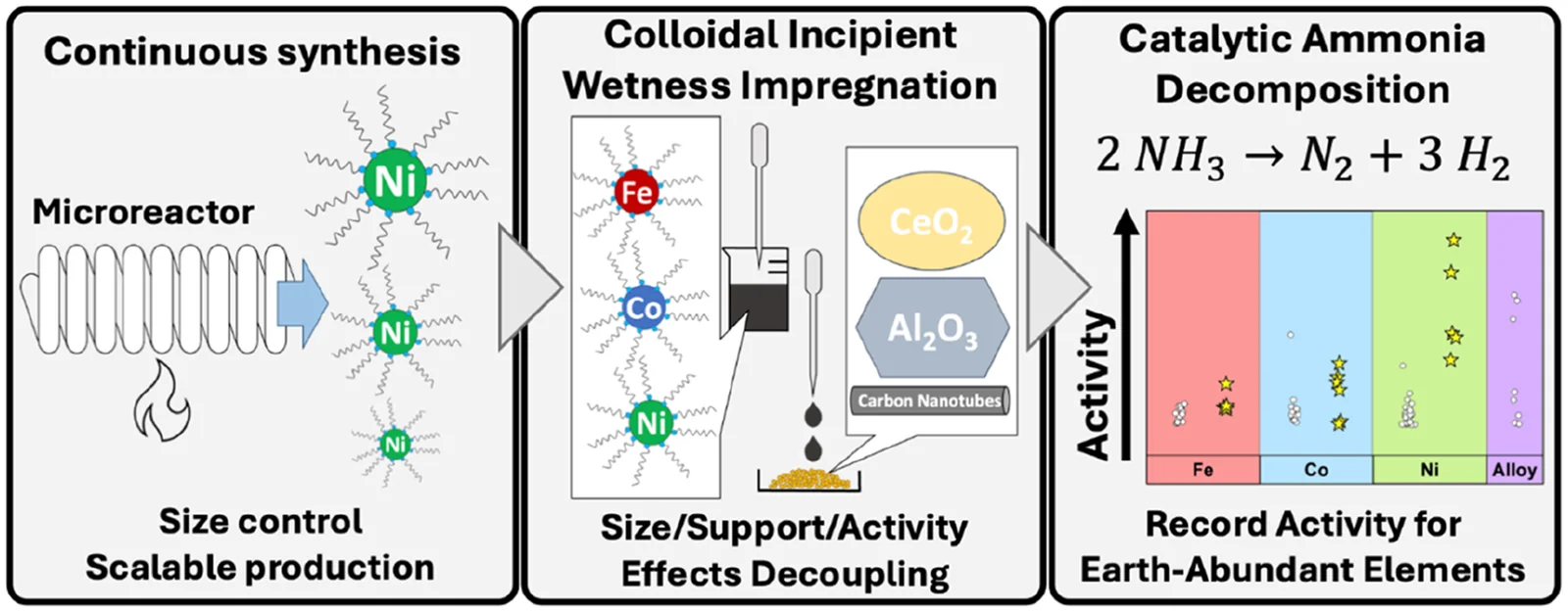

## Introduction

The rapid change in our climate, caused by our reliance on fossil fuels to drive our lifestyles and economy, is one of the most significant challenges humanity faces in the 21st century. The transition towards a de-fossilised society depends upon the use of renewable energy sources to meet our diverse energy demands. However, there is a generation-demand mismatch because of the intermittent nature of renewable energy.^1^ Long-term storage of renewable energy in chemical bonds is one of the most attractive solutions to overcome the interannual variations in renewable energy. In this context, green ammonia is at the forefront of renewable energy carriers because of its high energy density, easy storage and existing transport infrastructure.^2–4^ In addition, ammonia as an energy carrier can be used in different ways suitable for a range of applications, such as the generation of electrical and mechanical energy. In most cases, total or partial cracking of ammonia into hydrogen is required.^4,5^ For example, in the case of fuel cells, total ammonia cracking is needed for the production of hydrogen^6^ while in combustion engines, partial ammonia cracking decreases NO_*x*_ emissions by using ammonia/hydrogen mixtures.^7^ Due to its practical potential, ammonia cracking, also referred as the thermal catalytic decomposition of ammonia, is currently a very active area of research.^8^2NH_3_ ⇌ N_2_ + 3H_2_State-of-the-art catalysts in the literature are normally based on ruthenium, both in terms of higher rates of reaction and low temperature activity.^9,10^ The high activity of ruthenium-based catalysts is based on their optimum nitrogen bonding energy, which enables the activation of the ammonia molecules while minimising poisoning by nitrogen adatoms, especially at low temperatures.^11,12^ Indeed, combining metals from both sides of the Sabatier plot^13^ has been shown to mimic ruthenium activity, for example: Co–Re alloys.^14^ Despite the progress in this field, the scarcity and high cost of ruthenium have shifted the attention towards the development of non-noble metal catalysts for ammonia cracking with a focus on iron, cobalt, nickel and alkali metal amides.^15–17^

Ammonia cracking is known to be a structure sensitive reaction. In Ru-based catalysts, the optimum size is estimated to be between 3–5 nm where the concentration of B5 active sites is maximised.^18,19^ Similarly, the activity of non-noble cobalt, iron and nickel active sites is believed to be strongly dependent on the size;^20–22^ however, the size control of earth-abundant metals still remains a challenge in catalysis.

A range of wet chemistry methods have been developed over the last decades for the synthesis of nanoparticles (NPs) where different capping ligands and surfactants are used to accurately control the particle sizes.^23^ A particularly interesting approach to achieve narrow size distribution of non-noble metal nanoparticles is the thermal decomposition of metal precursors in organic solvents.^24,25^ Despite their potential, the batch nature of these approaches makes their scale up and reproducibility challenging, which hinders their deployment in the field of catalysis where large quantities of material are required.

Guided by fluid dynamic simulations, our group has developed flow 3D reactors for the controllable synthesis of metal nanoparticles.^19,26–29^ By promoting the formation of Dean vortices in helical reactors, Lagrangian mixing is enhanced to minimize the boundary layer in microreactors, resulting in the homogeneous growth of particles and leading to narrow size distributions.^30–33^ We have recently demonstrated this approach for the continuous flow synthesis of metal iron nanoparticles *via* thermal decomposition and PEG-functionalised iron oxide nanoparticles *via* co-precipitation for biomedical applications.^33,34^

In this paper, we develop highly active earth-abundant based catalysts for ammonia cracking by supporting pre-synthesised nanoparticles *via* colloidal incipient wetness impregnation (CIWI). Highly concentrated hydrocarbon ligand-stabilised iron, cobalt and nickel are synthesised in flow with *in situ* stabilisation using long-chain hydrocarbon organic ligands followed by their deposition onto a range of supports. We demonstrate that the presence of ligands not only enables the synthesis of small (<10 nm), homogeneous and stable nanoparticles, but they also contribute to the reduction of non-noble metals at lower temperatures, reducing their susceptibility towards agglomeration. The accurate size control achieved herein leads to the most active earth-abundant catalyst for the ammonia cracking reaction reported to date while providing insights into their activity–size relationships.

## Experimental section

### Continuous synthesis of colloidal metal nanoparticles

The continuous flow synthesis of iron, cobalt and nickel nanoparticles was carried out in a custom-made glass helical reactor consisting of a coiled 2 m glass capillary tube with 1 mm inner diameter and 6 mm outer diameter. The helical configuration had an external 24 mm diameter. Full details of the reactor set-up can be found somewhere else.^33^ The reactor was immersed in a silicone oil bath heated to the desired reaction temperature ranging from 170 to 200 °C. The temperature of the oil bath was controlled *via* a thermocouple connected to a hot plate. A solution of the precursor and long-chain hydrocarbon organic ligands (such as oleylamine, oleic acid and trioctylamine) was prepared by dissolving the required amount of the metal precursor into an argon-sparged solution of the ligands in the desired solvent. Various combinations of precursors, ligand concentration, temperature and flow rates were used to obtain the different metal nanoparticles of controlled sizes, which are detailed in Table 1. The respective precursors for Fe, Co and Ni were iron(0) pentacarbonyl (Fe(CO)_5_), dicobalt(0) octacarbonyl (Co_2_(CO)_8_), and nickel(0) dicyclooctadiene (Ni(COD)_2_), all purchased from Sigma Aldrich. The resulting precursor solution was loaded into a syringe through standard air-free techniques and connected to the glass reactor. To ensure oxygen-free conditions, the reactor was continuously flushed with argon while it was heated to the desired reaction temperature. Once heated, the precursor solution was pumped into the reactor with a syringe pump (Harvard 11 Elite). A volume of the solution equivalent to two reactor volumes (3 mL) was allowed to flow through the reactor before the product solution was collected, to ensure a fully developed steady state flow. The outlet of the reactor was collected into a flask containing a stabilizing ligand to ensure full stability of the resulting colloidal suspensions as specified in Table 1.

**Table 1:** Summary of the synthesis conditions for various nanoparticle metals and sizes

Sample ID	Precursor	Precursor concentration (mM)	*T* (°C)	Solvent	Ligand(s)	Metal : ligand(s) molar ratio	Ligand in collection flask	Flowrate (mL min^−1^)	Size (nm)
Fe NP_4.4 nm	Fe(CO)_5_	260	200	Octadecene	Oleylamine	1 : 1	Oleic acid	2	4.4 ± 1.2
Fe NP_10.1 nm	Fe(CO)_5_	260	200	Octadecene	Oleylamine	1 : 0.18	Oleic acid	1	10.1 ± 0.8
Co NP_2.4 nm	Co_2_(CO)_8_	73	170	Trioctylamine	Oleic acid	1 : 0.11	Oleylamine	2	2.4 ± 0.4
Co NP_10.7 nm	Co_2_(CO)_8_	73	170	Dodecane	Trioctylamine, oleic acid	1 : 0.84 : 0.11	Oleylamine	0.5	10.7 ± 1.4
Ni NP_2.4 nm	Ni(COD)_2_	100	170	Octadecene	Oleic acid	1 : 0.1	Oleic acid	0.5	2.4 ± 0.3
Ni NP_3.0 nm	Ni(COD)_2_	100	170	Octadecene	Oleic acid	1 : 0.025	Oleic acid	0.5	3.0 ± 0.8

After synthesis, the resulting colloidal suspensions were purified by the addition of acetone as an antisolvent to precipitate the ligand-stabilised nanoparticles. The mixture was then centrifuged at 1000*g* for 30 min. The supernatant was discarded and the precipitated nanoparticles were re-suspended in a minimum volume of *n*-hexane. The purification step was repeated three times.

Transmission electron microscopy images of the samples were acquired using a FEI Tecnai F20 G2 200 kV FEGTEM with a Gatan image filter (GIF) 200 and a 4k × 4k CCD detector. HAADF-STEM images and STEM-EDX elemental maps were acquired at 200 kV on a FEI Osiris STEM equipped with a Bruker Super-X quadruple EDS detector. Hexane colloidal suspensions of Fe, Co and Ni nanoparticles were drop-cast onto on a Cu-supported ultrathin carbon membrane (Agar Scientific). For particle size distribution determination, at least 100 particles taken from 3 or more representative images were counted and measured for each sample using ImageJ software.

### Preparation of catalysts by colloidal incipient wetness impregnation (CIWI)

The concentrated colloidal suspensions of Fe, Co and Ni nanoparticles were deposited in commercial supports, CeO_2_ (nanopowder <25 nm, Sigma Aldrich), γ-Al_2_O_3_ (nanopowder, Alfa Aesar) or carbon nanotubes (multiwall, Nanocyl), dropwise *via* incipient wetness impregnation of the concentrated colloidal suspensions in hexane. The incipient wetness volume of the different supports was measured using the same solvent. The impregnated powders were then dried in a vacuum oven at 80 °C for 2 hours.

The metal loading of the different catalysts was determined using inductively coupled plasma mass spectroscopy (ICP-MS, NexION 5000, Perkin-Elmer). For that, a known amount of catalyst was digested in freshly prepared *aqua regia* at 80 °C for 12 hours before measuring its metal content. Temperature programmed reduction (TPR) experiments were carried out using a Micromeritics AutoChem II equipped with a thermal conductivity detector. The analyses were performed using 30 mL min^−1^ of 5% H_2_/Ar with a ramp rate of 10 °C min^−1^. The gases produced during the measurement were analysed using a mass spectrometer (MKS Cirrus 2).

### Preparation of reference Ru/CeO_2_ catalysts by incipient wetness impregnation

A reference 3 wt% ruthenium on ceria catalyst was prepared by incipient wetness impregnation (IWI) of a ruthenium nitrosyl nitrate solution into ceria. The required amount of the metal precursor was dissolved in the respective wetness volume of water and CeO_2_ was impregnated dropwise. The impregnated powder was dried in a vacuum oven at 80 °C for 2 hours.

### Ammonia cracking catalytic tests

Ammonia decomposition reactions were carried out in a continuous differential packed bed reactor. The packed bed was prepared in a quartz U-tube using 25 mg of catalyst diluted in around 400 mg silicon carbide bed (46 grit) to achieve a 4 cm^3^ bed volume. Before the catalyst bed, a 200 mg buffer of fine silicon carbide (250–400 mesh) was placed to ensure an even gas flow. The reactor system was equipped with mass flow and temperature controllers. The thermocouple was placed right above the catalytic bed to avoid any temperature lag. All the pipes were heated to 60 °C to avoid ammonia condensation and corrosion. When specified, the catalysts were pre-calcined *in situ* under a flow of O_2_ 5% in argon (10 mL min^−1^) at 300 °C for 2 hours. In all cases, the catalysts were reduced *in situ* under pure H_2_ flow (10 mL min^−1^) at 600 °C for 30 minutes. The temperature was then reduced to room temperature under the H_2_ flow. Following this, the reaction temperature was ramped from room temperature to 600 °C using a Carbolite tube furnace equipped with a PID controller. A 2.6 °C min^−1^ ramp under 2.5 mL min^−1^ NH_3_ and 6 mL min^−1^ He (GHSV of 6000 mL_NH_3__ g_cat_^−1^ h^−1^) was used. Three temperature cycles up to 600 °C and down to 200 °C were carried out in each catalytic test. The reactor exit gas was analysed using an on-line gas chromatograph (Agilent) fitted with a Hayesep Q column and equipped with a thermal conductivity detector. Mass balance calculations were carried out to account for the molar expansion occurring as a result of the reaction. Mass balance was achieved within a ±10% error.

## Results and discussion

Heterogeneous non-noble metal-based catalysts were developed by colloidal incipient wetness impregnation (CIWI) of highly concentrated colloidal nanoparticles. The method consists of two main steps (see Fig. 1). Firstly, highly concentrated colloidal nanoparticles with narrow size distributions are synthesised in a flow reactor by the thermal decomposition of zero-valent metal precursors in the presence of long chain hydrocarbon organic capping ligands such as oleic acid, oleylamine and trioctylamine, acting as stabilisers. Then, the resulting nanoparticle colloidal suspensions are deposited on supports by their incipient impregnation facilitated by the high metal concentration of the organic suspension of nanoparticles. In contrast to the standard incipient wetness impregnation (IWI) method, in this approach, nanoparticles are directly supported rather than their metal precursors. This approach enables precise control on the particle size prior to their deposition on any desired support. This method departs from previously reported ones where colloidal nanoparticles were deposited by adsorption in the support, typically resulting in poor control over the metal loading and the need for additional purification steps.^35,36^ Instead, the CIWI allows high loading rates for cobalt, iron and nickel nanoparticles, demonstrated herein for the development of supported catalysts for ammonia cracking for the production of hydrogen.

**Fig. 1:**
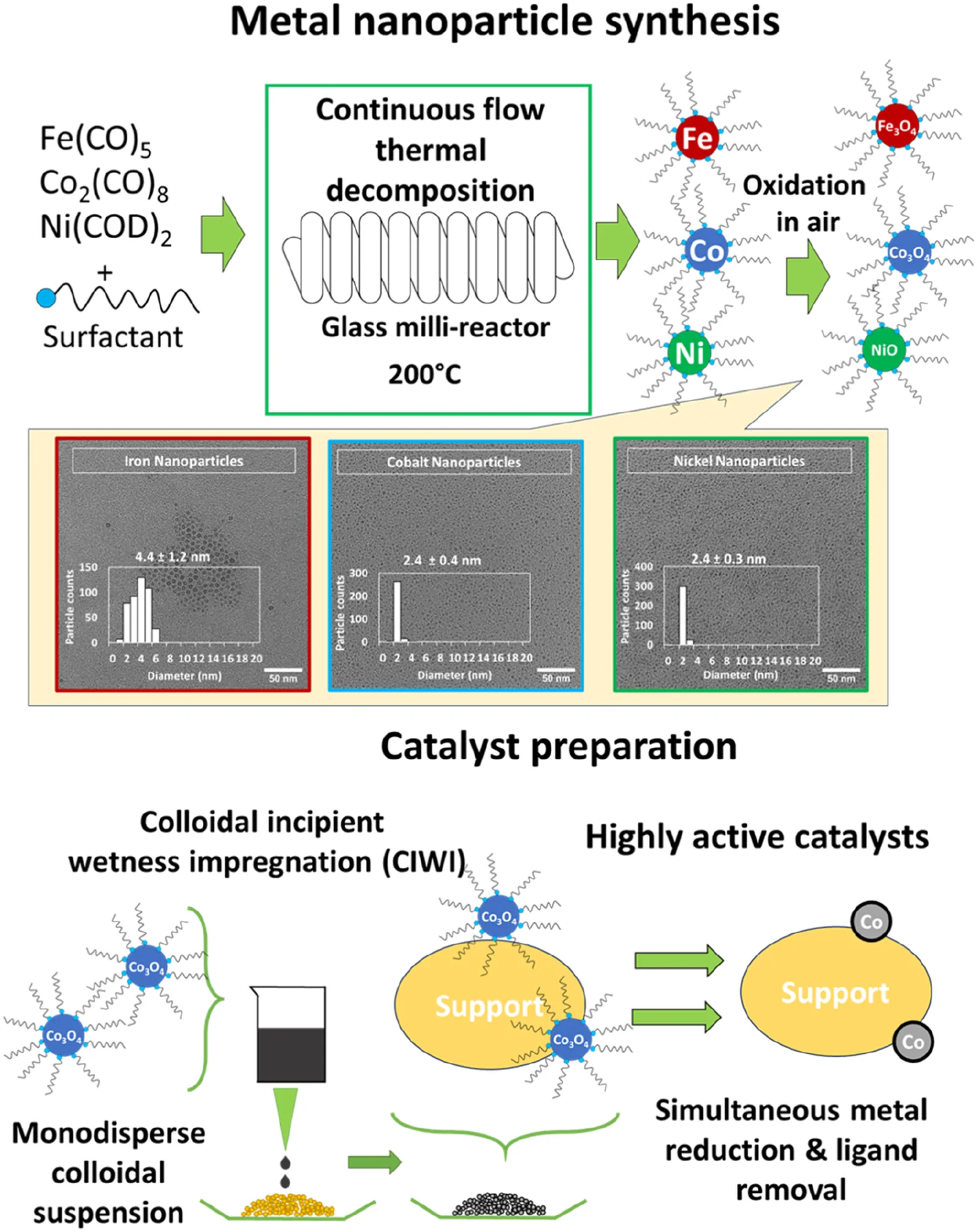
Schematic of the colloidal incipient wetness impregnation (CIWI) method for the synthesis of catalysts. In the first step, highly concentrated suspensions of metal nanoparticles are synthesised in flow reactors with controlled sizes and narrow size distribution. In the second step, the nanoparticle suspensions are impregnated in supports. TEM pictures show representative examples of cobalt, iron and nickel nanoparticles respectively, their size distribution and synthetic conditions.

This method of synthesising metal nanoparticles by thermal decomposition of their zero-valent precursor was originally developed for iron nanoparticles^33^ and adapted herein to cobalt and nickel nanoparticles using appropriate precursors and ligands. A range of different nanoparticle sizes are achieved by varying the reaction conditions such as temperature, solvent, flow rate and organic capping ligand concentration, as shown in Table 1.

The different ligands used can potentially displace the carbonyl or dicyclooctadiene group in the metal precursor compounds, forming complexes with intricate equilibrium. Such equilibrium can be affected by the chemical environment (*e.g.* solvent and metal : ligand molar ratio). The rate of thermal decomposition of each of these complexes would determine the nucleation and growth rate of the metal, ultimately affecting the resulting metal nanoparticle size. In general, a higher reaction temperature, a lower metal : ligand ratio and a more coordinating solvent lead to smaller nanoparticles because of an increased nucleation rate and stabilization of a higher nanoparticle surface area. A microreactor system with small channel diameters enables fast (<a few seconds) heating of the reaction medium, leading to a homogenous nucleation environment.^28^ In addition, the 3D helical configuration of the reactor promotes the rotation of the fluid, promoting gentle Lagrangian mixing, flattening the conventional parabolic profile of microreactors, and leading to narrow residence times.^31,37^

The resulting nanoparticle suspensions are purified by a series of precipitation–centrifugation steps (see Experimental section) and stored in hexane, showing a very high stability *versus* agglomeration over several months. During the purification, the particles are exposed to air, leading to their rapid oxidation from the original metallic state to an oxide.^38,39^ Representative TEM micrographs are shown in Fig. 2. In the case of larger nanoparticles (>10 nm), a core–shell structure is visible, because of the self-limiting oxidation with a maintained metallic core. In all cases, the nanoparticles are amorphous, not displaying any lattice fringes during HRTEM characterization. The amorphous state is likely due to the rapid nucleation and growth conditions achieved during the thermal decomposition reaction.^38^

**Fig. 2:**
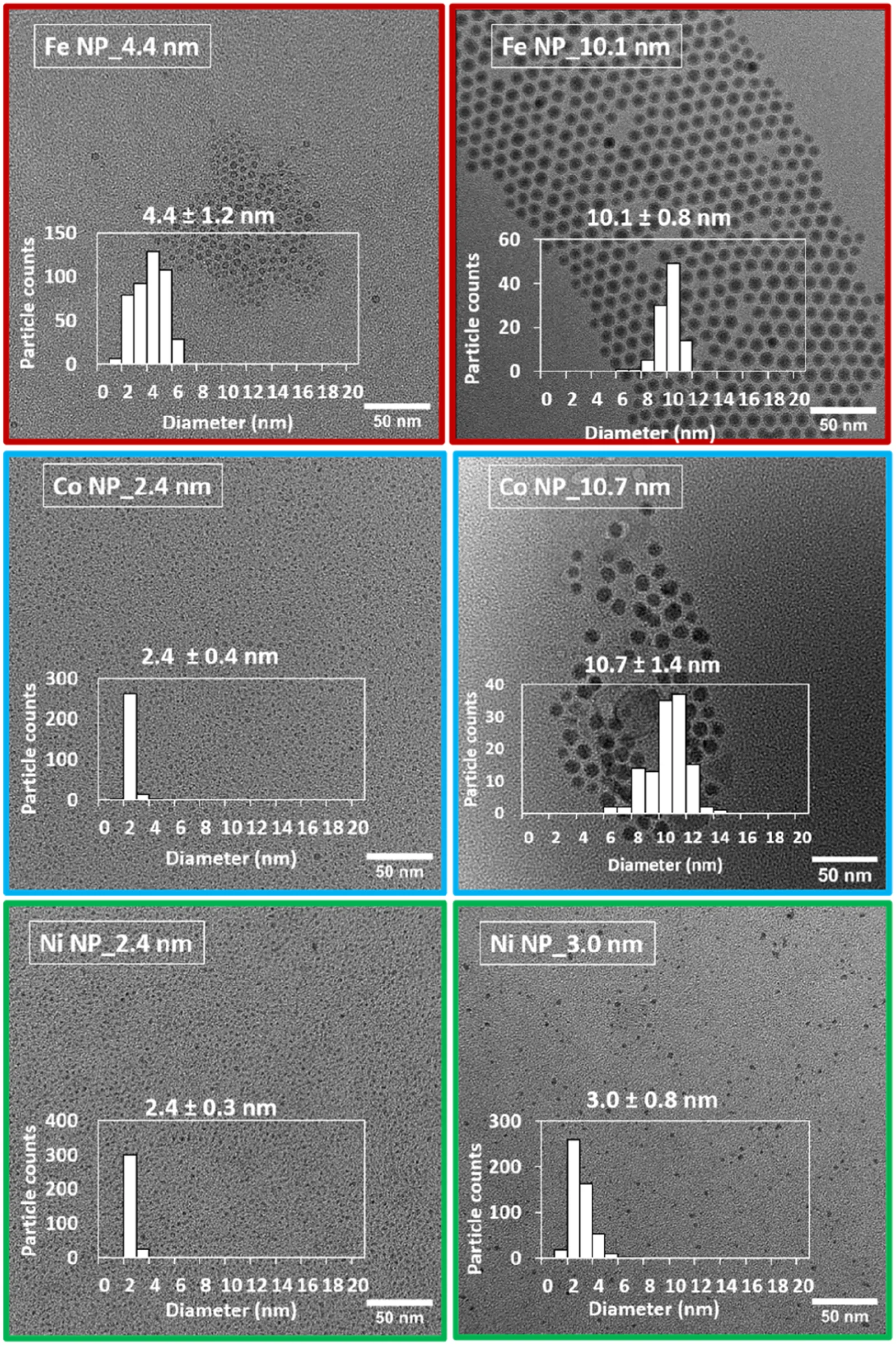
Representative TEM images and particle size distribution of iron, cobalt and nickel nanoparticles synthesised *via* thermal decomposition in a continuous flow reactor. Reaction conditions (surfactants, metal : ligand ratios, concentrations, solvent, temperature and flow rate) are shown in Table 1.

The colloidal nanoparticles are supported by colloidal incipient wetness impregnation on CeO_2_ and their catalytic activity for ammonia cracking for hydrogen production is tested. The catalytic studies are carried out under dynamic temperature conditions during three consecutive cycles. In each cycle, the temperature is ramped from 200 °C to 600 °C and then lowered back again to 200 °C. All catalysts are reduced *in situ* under a 10 mL min^−1^ of H_2_ flow for 30 min at 400 °C. When specified, the catalysts are also calcined prior to reduction under a 5% O_2_/He 10 mL min^−1^ flow for 2 h at 300 °C.

Fig. 3a shows that the ammonia cracking activity decreases as Ni > Co > Fe, which is consistent with the trend reported in the literature^15,40^ although this ranking order sometimes depend on the reaction conditions.^15^ Iron and cobalt-based catalysts seem to have weak size-activity dependency as similar activities are obtained with particles between 2–4 nm and >10 nm. It is important to note that in the case of cobalt nanoparticles, a re-structuring effect is observed under reaction conditions leading to ∼6–7 nm particle sizes after the reaction independently of the starting size (see below). Despite the reversibility principle between ammonia synthesis and the decomposition reaction, iron-based catalysts, universally used in ammonia synthesis, show low activity for the ammonia cracking reaction due to the high nitrogen binding energy that makes iron prone to nitridation.^41^ In contrast, the low nitrogen binding energy of nickel makes it particularly active under the studied reaction conditions. In addition, nickel nanoparticles show a strong size–activity effect as 2.4 nm nanoparticles present a 3-fold activity increase compared to 3.0 nm particles. Nickel is known to exhibit structure-sensitive behavior in this reaction as previously observed experimentally and *via* DFT calculations.^42^ The (110) facet is more active than the (111) and (211) facets; therefore the geometric and electronic structure of Ni catalysts is strongly determined by the Ni NP size and shape. The optimal particle size has been identified to be around 2–3 nm in agreement with our observations.^43^ For comparison, a Ru-based catalyst is prepared by incipient wetness impregnation, showing an activity at 500 °C of ∼5005 mol_NH_3__ mol_metal_^–1^ s^–1^, *versus* the ∼3130 mol_NH_3__ mol_metal_^–1^ s^–1^ of the Ni-based catalysts. Considering that Ni is ∼1000 cheaper than Ru, this novel approach presents a unique potential for the synthesis of size-controlled earth-abundant metal nanoparticles as catalysts for ammonia cracking.

**Fig. 3:**
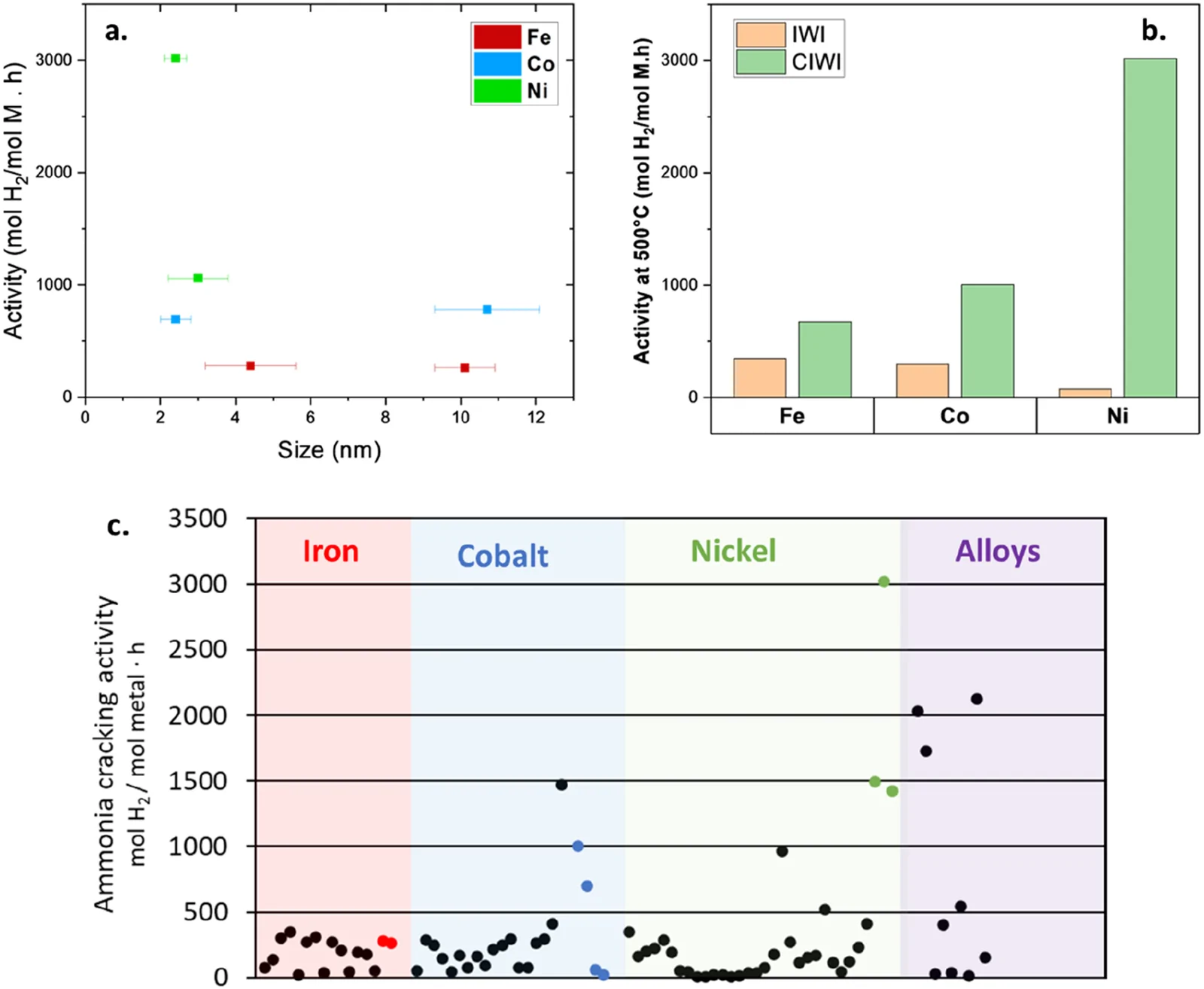
a. Size–activity relationship of catalytic ammonia decomposition activity of Co, Ni and Fe NPs supported by CIWI on CeO_2_ at 500 °C. b. Comparison of the CIWI catalysts with those prepared by the conventional incipient wetness impregnation (IWI) method.^9,44,45^ c. Comparison of the catalytic data in this work (red, blue and green dots) with the state-of-the-art catalysts in the literature (black dots) at 500 °C, shown as the apparent rate of reaction. Details of the comparison and literature review are included in Table S1 in the SI.

In order to compare the activity of the different catalysts, as well as those reported in the literature, the apparent rate of reaction (mol_H_2__ mol_metal_^–1^ h^–1^) was calculated at 500 °C. At this temperature, the conversion level is <40% and thus the values are believed to be not affected by mass transfer limitations or equilibrium. The catalysts prepared by the CIWI present an appreciable increase in activity in comparison to catalysts prepared by the conventional incipient wetness impregnation of metal precursors as shown in Fig. 3b. The difference is attributed to the exceptional size control of conventionally difficult to control earth-abundant metal nanoparticles (cobalt, nickel and iron) that the CIWI method achieves. Because of the homogenous particle sizes achieved, this method leads to homogeneous active site structure and properties, increasing the atom efficiency of the catalysts. The relevance of the CIWI method is further highlighted by comparing the activity of these catalysts with the state-of-the-art non-noble metal catalysts reported in the literature. Fig. 3c shows the high activity achieved with this method in comparison to other methods employed in the literature including incipient wetness impregnation, co-precipitation, hydrothermal synthesis, *etc.* In particular, the ammonia cracking activity of the small 2.4 nm nickel nanoparticle catalyst prepared by the CIWI reported herein shows the highest catalytic activity when compared to the best reported non-precious metal based catalysts in the literature, surpassing that of CoMo alloys^46,47^ or high entropy CoMoFeNiCu catalysts.^48^ The complete data of the review of the catalyst literature is compiled in Table S1 in the SI. This comparison demonstrates the advantages of using long-chain hydrocarbon ligands for the synthesis of highly active catalysts, which can be applied to other reactions of interest.

The activity of the non-calcined catalysts increases significantly after the first ammonia cracking cycle when the temperature is increased from ∼200 °C to 600 °C as shown in Fig. 4 for the nickel-based catalyst and Fig. S5 for the other metals. After the first cycle, the activity of all the catalysts remains constant in consecutive cycles and almost equal to the activity presented by the pre-calcined catalysts (Fig. S6), suggesting the removal of the ligands during the reaction.

**Fig. 4:**
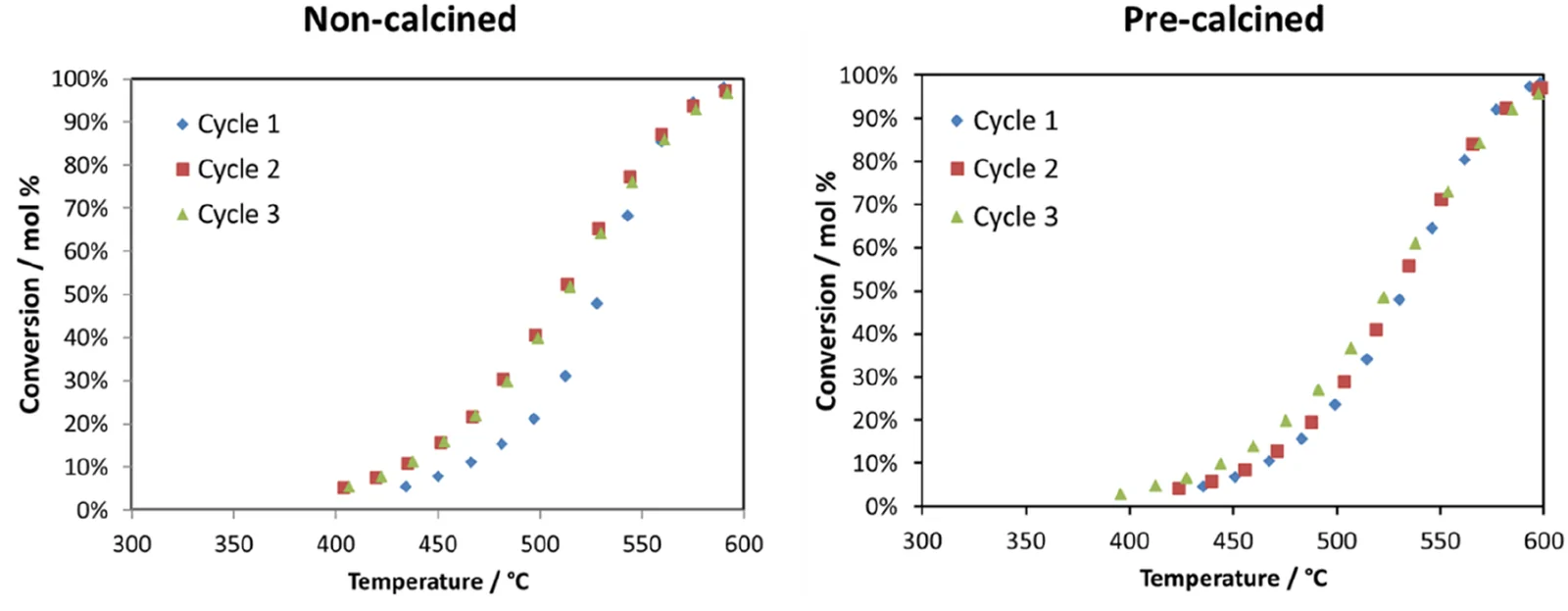
Ammonia decomposition catalytic activity of nickel nanoparticles (2.4 nm) supported on CeO_2_ by the CIWI method as a function of temperature in consecutive runs in a. non-calcined catalyst and b. pre-calcined catalyst under 5% O_2_/He 10 mL min^−1^ flow for 2 h at 300 °C. Details of synthetic conditions of the nickel nanoparticles are given in Table 1.

While it is generally accepted that capping ligands have negative effects on the catalytic activity of metal nanoparticles because of steric repulsions and coordination to the active sites,^49^ herein, we demonstrate the beneficial effect of long chain hydrocarbon ligands to facilitate the reduction of the metal active sites while avoiding nanoparticle sintering. To fully elucidate the fate of the ligands, calcined and non-calcined catalysts are analysed using temperature programmed reduction under H_2_ flow (H_2_-TPR) with the outlet gases analysed by mass spectrometry (Fig. 5).

**Fig. 5:**
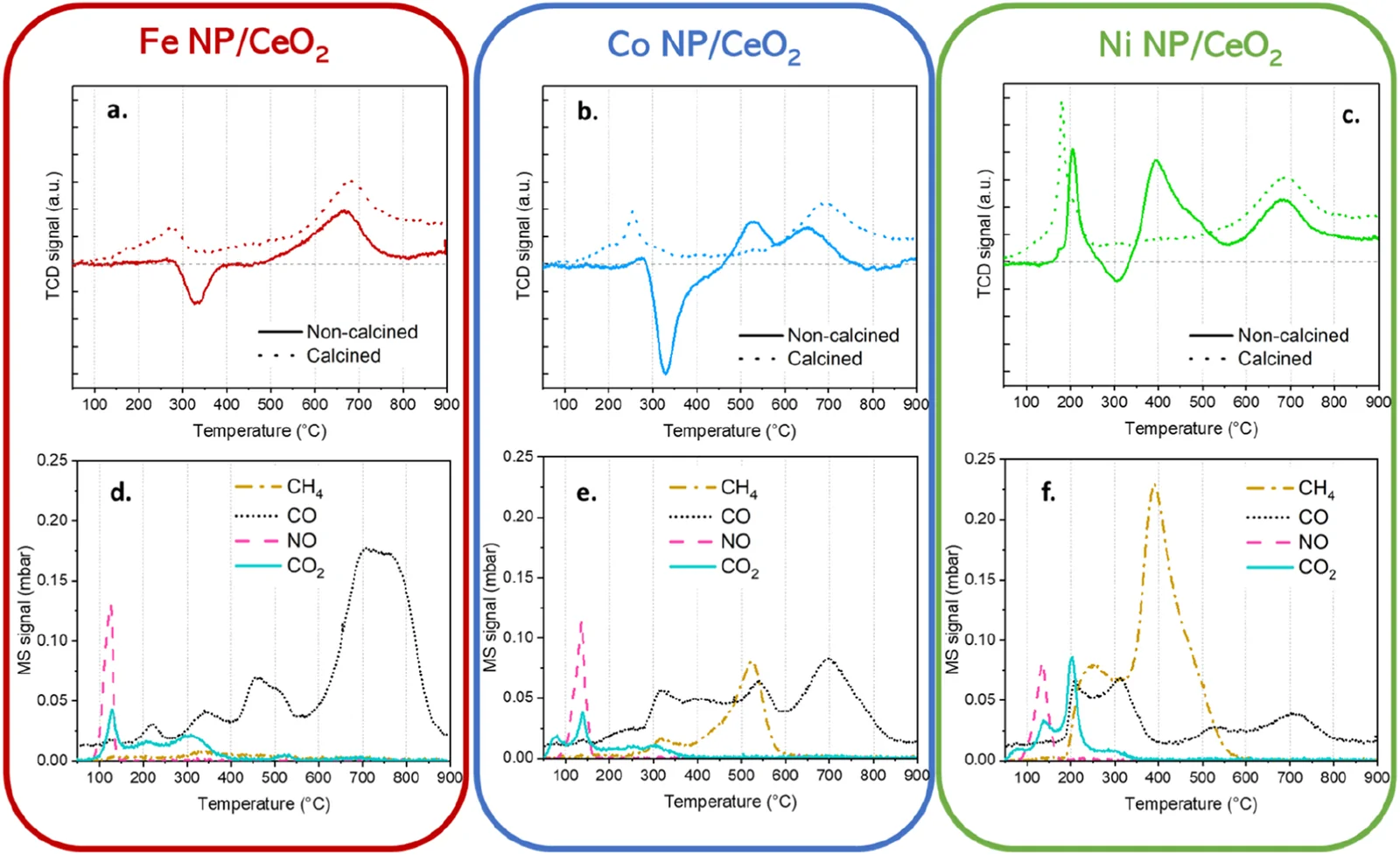
a–c) H_2_-TPR profiles of various catalyst samples on CeO_2_, reduced up to 900 °C without calcination (continuous line) and with preliminary calcination (dashed line). d–f) MS spectra of the gases produced from the organic ligands decomposition during the H_2_-TPR of the non-calcined catalysts. The initial particle sizes for Fe, Co and Ni were 4.4 ± 1.2, 10.7 ± 1.4 and 2.4 ± 0.3 nm, respectively. No evolved gases were detected in MS during H_2_-TPR of calcined catalysts.

Calcination of the samples is carried out at 300 °C under a 5% O_2_ flow, leading to the removal of the organic ligands *via* their oxidation into water, CO and CO_2_. The H_2_-TPR of the calcined samples presents the corresponding reduction peak of the different metals (Ni at 187 °C, Co at 263 °C and Fe at 270 °C). The broadness of the metal reduction peak follows that of the metal size distribution and matches that measured by TEM (Fig. 2). Regarding the ceria support, two characteristic reduction peaks are present as shown in Fig. S1 (top) for pure CeO_2_ support, corresponding to the surface and bulk oxygen respectively.^50^ In the three cases, the first surface oxygen reduction peak seems to overlap with the reduction of the metal peak, suggesting a strong metal–support interaction and reason of the lower reduction temperature of the metals in comparison to the literature (Ni: 200–250 °C,^51^ Co: 280–390 °C,^52^ Fe: 375–500 °C (ref. 53)).

A mixture of gases is released during the H_2_-TPR analysis of the non-calcined catalysts including H_2_, NO, CO, CO_2_ and CH_4_ (Fig. 5) The CO_2_ and NO released at low temperatures (∼100 °C) is associated to the impurities in the commercial ceria (Fig. S1, top), likely Ce(NO_3_)_3_ and Ce_2_(CO_3_)_3_. The negative peak observed at ∼300 °C is associated to H_2_ generation as confirmed by carrying out the analysis under pure argon flow (Fig. S2). The negative TCD signal is associated to the higher thermal conductivity of hydrogen gas compared to the helium carrier gas. Such H_2_ generation is coupled with CO generation (as observed by mass spectroscopy), which can be associated to the sequential reduction of the metal oxides by the hydrocarbon ligands (and thus partial oxidation of the ligands to CO) followed by subsequent catalytic dehydrogenation of the ligands (eqn (1) and (2) respectively). Note that in these reactions, the ligands are represented by the general formula of an unsaturated aliphatic compound, representative of oleic acid and oleylamine used herein.1
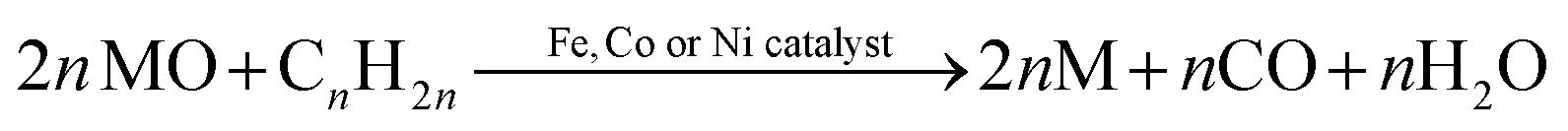
2
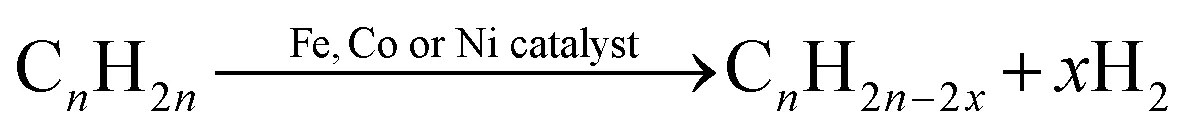
Alternatively, it is also possible that a combination of these two reactions occurs, in a process akin to a reverse Fischer–Tropsch reaction (eqn (3)).3
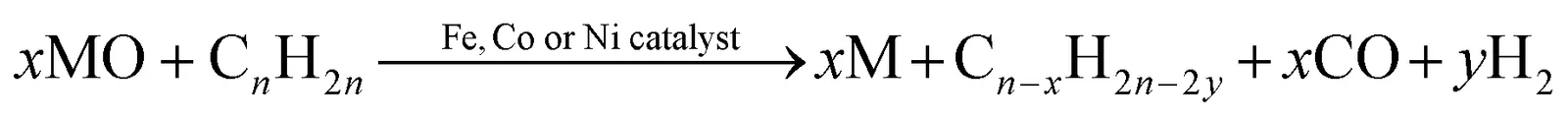
The release distribution of the different gases as a function of temperatures indicates that the first pathway is dominant, as CO and H_2_ are not released at the same temperatures, but it is unclear whether the second pathway is also happening. It is possible that the CeO_2_ supports contribute to these pathways, facilitating the oxidation processes with oxygen from its crystal lattice.^54^

In addition, CH_4_ is released in the Co and Ni catalysts because of the methanation of the ligands according to eqn (4). This is a well-known effect in the Fischer–Tropsch literature and one of the reasons why Ni-based catalysts are avoided in Fisher–Tropsch synthesis.^55^ The methanation of the ligands takes place at lower temperatures for Ni (∼400 °C) than Co (∼500 °C). In addition, the amount of methane release in the Co-based catalyst is considerably lower than in the case of Ni and is inexistant for the Fe-based catalyst, consistent with the methane selectivity reported in the literature for those metals.^56^4
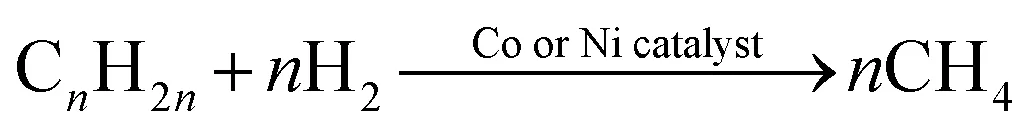
In all catalysts, CO is also generated between 600–900 °C. This peak is associated to the degradation of any remaining organic compounds including ligands. It is interesting to note that the extension of CO generation is inversely proportional to the formation of methane at lower temperatures. From these observations, one can infer that methanation is an important route for ligand removal in these catalysts. As methanation does not take place in the iron-based catalysts, their ammonia cracking activity is greater in pre-calcined catalysts than in non-calcined ones. In contrast, those metals with a high methanation activity (*e.g.* Ni and Co) present similar activities in the pre-calcined catalysts and the non-calcined ones after the first catalytic cycle as the ligands are fully removed at 600 °C under reaction conditions (H_2_ is released by NH_3_ cracking) (Fig. 4).

In most cases, the metal nanoparticle size is maintained during the calcination, reduction and reaction as demonstrated by electron microscopy. Because of the high electron contrast of the ceria support making metal nanoparticle identification difficult, HAADF-TEM combined with EDX elemental mapping was used to determined particle size distribution (Fig. 6). At least 100 particles were counted and measured based on the K-edge EDX images of the different metals. Fe and Ni-based catalysts conserve their original particle size after at least 3 catalytic cycles of ammonia cracking, with stable catalytic activity as shown above. In contrast, the Co-based catalyst is re-structured under reaction conditions leading to slightly smaller average particle sizes (10.7 nm in the fresh catalysts and 6.7 nm in the spent catalyst) and broader size distribution. A similar re-structuring, associated to the strong metal–support interactions on ceria supports, have been previously observed in the context of Fisher–Tropsch cobalt catalysts.^57^ It has been previously postulated that this type of restructuring can be induced by the presence of organic ligands.^58^

**Fig. 6:**
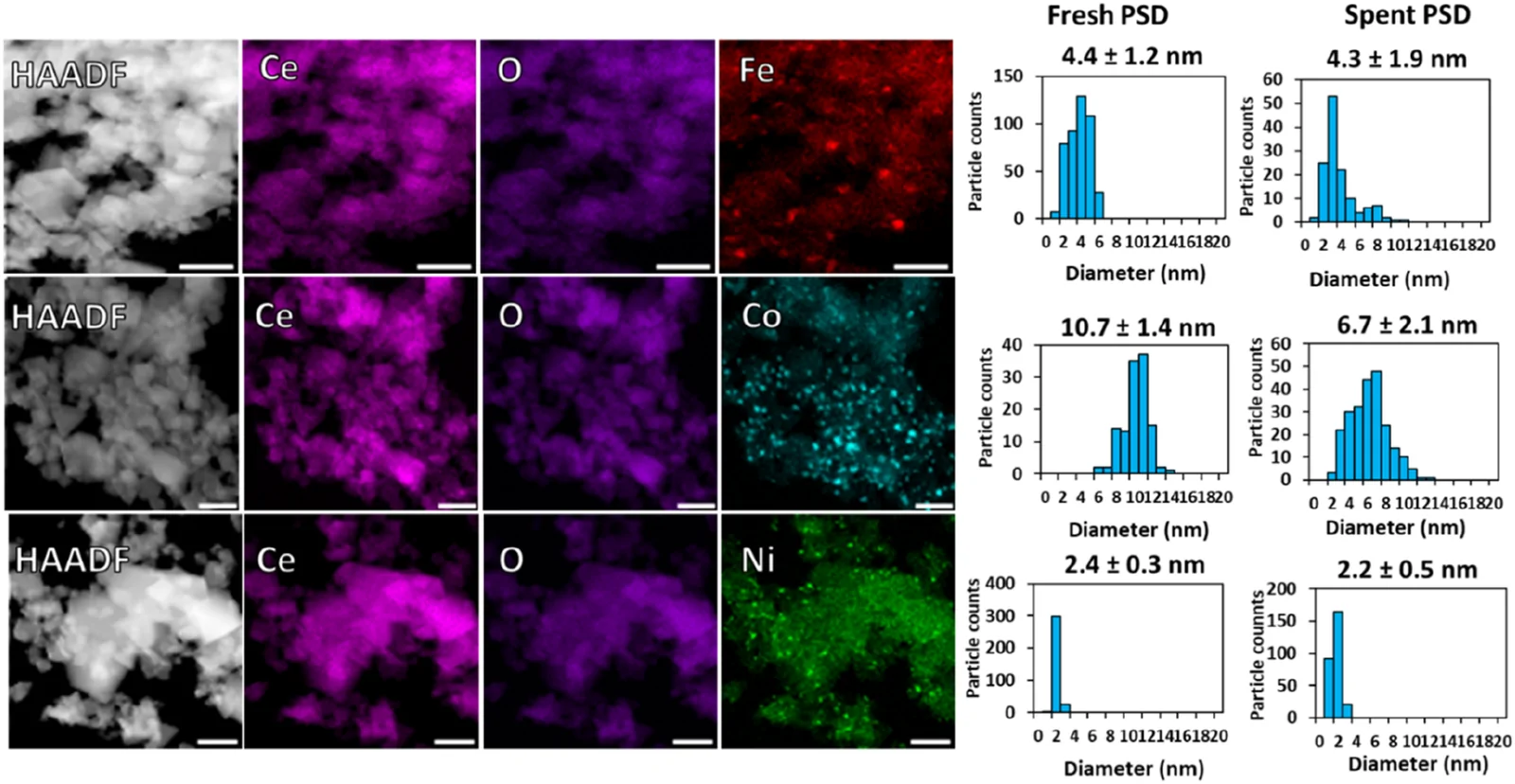
HAADF-STEM images and EDX elemental mapping of Fe, Co and Ni -based catalysts supported *via* the CIWI method on the CeO_2_ catalyst after 3 cycles of NH_3_ decomposition reaction. All scale bars are 50 nm. The corresponding calculated particle size distribution before (fresh PSD, left, calculated from TEM of the original colloidal suspension) and after the reaction (spent PSD, right, calculated form the respective K-edge EDX images) are shown for each catalyst. No calcination pre-treatment was applied to these three catalysts.

An additional advantage of the developed CIWI method is the possibility of decoupling of the size–support dependency. To demonstrate this, cobalt and nickel nanoparticles were deposited on different commercial supports including ceria, alumina and carbon nanotubes (CNTs) by the CIWI. Fig. 7 shows that the activity of the metal nanoparticles is strongly dependent on the nature of the support, with activity decreasing as CeO_2_ > Al_2_O_3_ > CNTs. The low activity of carbon-based supports is attributed to their methanation and thus degradation under reaction conditions, evidenced by a large methane peak observed by MS during H_2_-TPR (see Fig. S3), particularly relevant in the case of Ni-based catalysts because of its high methanation activity as discussed above.

**Fig. 7:**
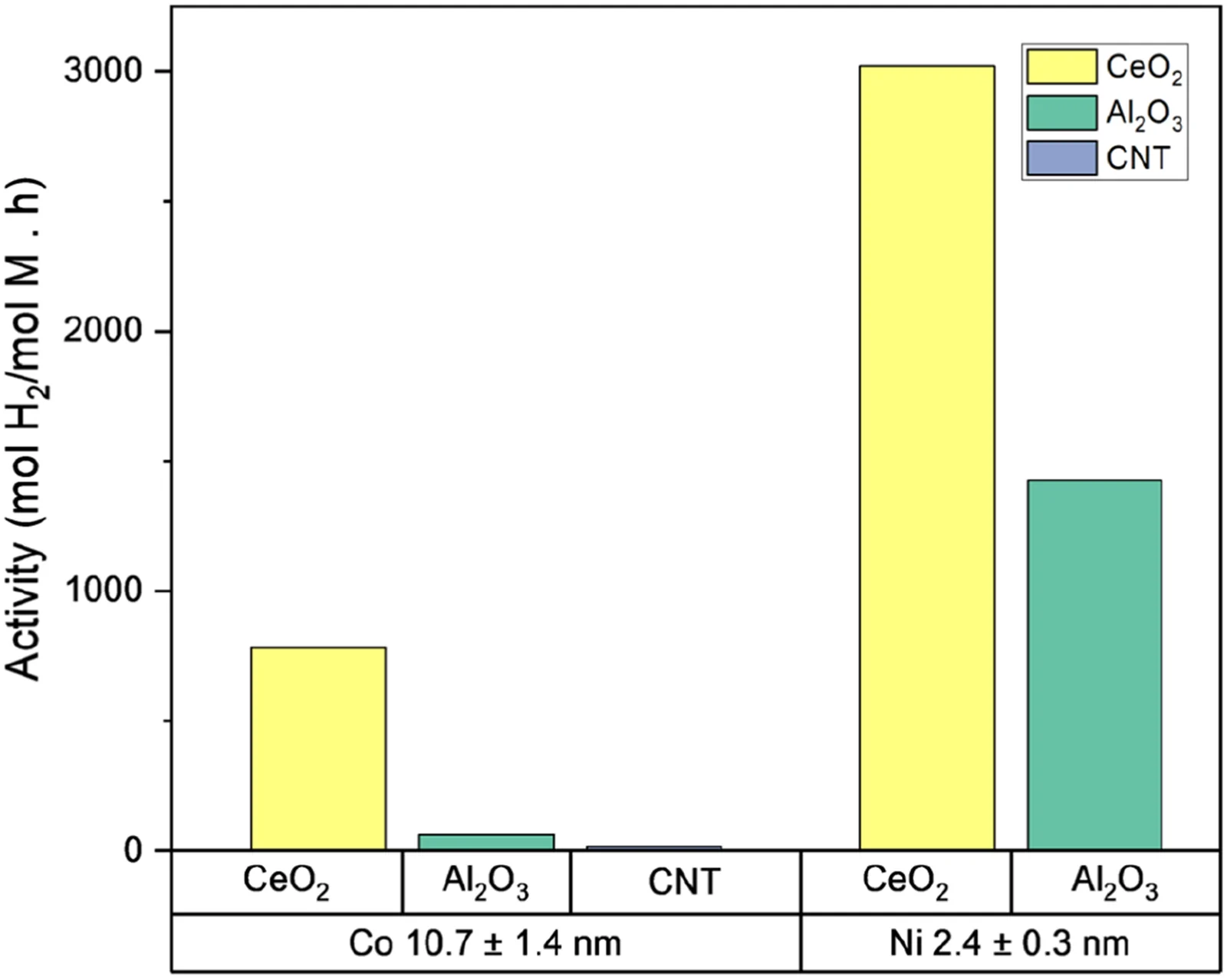
Effect of the support on the catalytic activity of cobalt (10.7 ± 1.4 nm) and nickel (2.4 ± 0.3 nm)-based catalysts at 500 °C.

While Ni nanoparticles supported on CeO_2_ presented a high stability with constant activity during consecutive catalytic cycles (as discussed above), the Ni-based catalytic activity decreased gradually when supported on Al_2_O_3_ (Fig. S7). Stability tests of the 2.4 nm Ni/CeO_2_ catalysts at 500 °C show an initial increase in activity likely to ligand removal followed by a steady decline in activity over 90 h. The higher stability of the ceria supported catalysts is not believed to be only attributed to the effect of the support on removing the ligands, but more likely to the stronger metal–support interaction in the ceria supported catalysts, which leads to a higher level of stabilisation against sintering, particularly at higher temperatures.^59–61^ In addition, ceria has recently been shown to also facilitate the electron transfer to the nickel active sites by optimising surface basicity.^62^

## Conclusions

This work presents the synthesis of highly active non-noble metal ammonia cracking catalysts by the colloidal incipient wetness impregnation (CIWI) of size-controlled colloidal Fe, Co, and Ni nanoparticles synthesised in flow reactors through thermal decomposition of zero-valent precursors. This approach offers a major improvement over conventional incipient wetness methods, overcoming issues related to poor control of metal dispersion and particle size. High colloidal concentrations of nanoparticles were stabilised by long-chain hydrocarbon ligands. *In situ* activation and ligand removal can be achieved without the need for high-temperature calcination, thanks to a self-cleaning behaviour under reaction conditions involving reductive and methanation pathways. The flexibility of the CIWI method allows for detailed investigation of size–activity relationships, revealing that the ammonia cracking activity of Ni-based catalysts is strongly size-dependent, while Fe and Co-based catalysts showed weaker size effects. Ni/CeO_2_ catalysts with 2.4 nm nanoparticles demonstrated exceptional activity for ammonia decomposition, outperforming previously reported earth-abundant catalysts and approaching the performance of Ru-based systems at a fraction of the cost. This new CIWI catalyst preparation method offers a powerful route to design and deploy non-noble metal catalysts with finely tuned properties and high catalytic efficiency, as demonstrated herein for hydrogen production from ammonia cracking and could be extended to a wide variety of different active metal/supports systems and target reactions.

## Author contributions

JM: conceptualization, data curation, formal analysis, investigation, methodology, writing – original draft, writing – review and editing; JE-K: data curation, investigation, writing – review and editing; LTM: conceptualization, funding acquisition, supervision, writing – review and editing.

## Conflicts of interest

On behalf of all authors, the corresponding author states that there is no conflict of interest.

## Supplementary Material

CY-OLF-D6CY01170E-s001

## Data Availability

The data supporting this article have been included as part of the supplementary information (SI). Supplementary information is available. See DOI: https://doi.org/10.1039/d6cy01170e.

## References

[cit1] Torrente-Murciano L., Smith C. (2023). Nat. Synth..

[cit2] David W. I. F., Agnew G. D., Bañares-Alcántara R., Barth J., Hansen J. B., Bréquigny P., de Joannon M., Stott S. F., Stott C. F., Guati-Rojo A., Hatzell M., Macfarlane D. R., Makepeace J. W., Mastorakos E., Mauss F., Medford A., Mounaim-Rousselle C., Nowicki D. A., Picciani M. A., Postma R. S., Rouwenhorst K. H. R., Sabia P., Salmon N., Simonov A. N., Smith C., Torrente-Murciano L., Valera-Medina A. (2024). JPhys Energy.

[cit3] T. R. Society , Ammonia: zero-carbon fertiliser, fuel and energy store, Policy briefing, 2020

[cit4] Schuth F., Palkovits R., Schlogl R., Su D. S. (2012). Energy Environ. Sci..

[cit5] Lamb K. E., Dolan M. D., Kennedy D. F. (2019). Int. J. Hydrogen Energy.

[cit6] Yin S. F., Xu B. Q., Zhou X. P., Au C. T. (2004). Appl. Catal., A.

[cit7] Valera-Medina A., Xiao H., Owen-Jones M., David W. I. F., Bowen P. J. (2018). Prog. Energy Combust. Sci..

[cit8] Saini P., Jampaiah D., Periasamy S., Kulkarni A. P., Bhargava S. K. (2025). Chem. Commun..

[cit9] Hu Z., Mahin J., Torrente-Murciano L. (2019). Int. J. Hydrogen Energy.

[cit10] Garcia-Garcia F. R., Alvarez-Rodriguez J., Rodriguez-Ramos I., Guerrero-Ruiz A. (2010). Carbon.

[cit11] Hill A. K., Torrente-Murciano L. (2015). Appl. Catal., B.

[cit12] Hill A. K., Torrente-Murciano L. (2014). Int. J. Hydrogen Energy.

[cit13] Jacobsen C. J. H., Dahl S., Clausen B. S., Bahn S., Logadottir A., Nørskov J. K. (2001). J. Am. Chem. Soc..

[cit14] Kirste K. G., McAulay K., Bell T. E., Stoian D., Laassiri S., Daisley A., Hargreaves J. S. J., Mathisen K., Torrente-Murciano L. (2021). Appl. Catal., B.

[cit15] Bell T. E., Torrente-Murciano L. (2016). Top. Catal..

[cit16] Zhu N., Yang F., Hong Y., Liang J. J. (2025). Int. J. Hydrogen Energy.

[cit17] Makepeace J. W., He T., Weidenthaler C., Jensen T. R., Chang F., Vegge T., Ngene P., Kojima Y., de Jongh P. E., Chen P., David W. I. F. (2019). Int. J. Hydrogen Energy.

[cit18] Garcia-Garcia F. R., Guerrero-Ruiz A., Rodriguez-Ramos I. (2009). Top. Catal..

[cit19] El-Kadi J., Torrente-Murciano L. (2026). React. Chem. Eng..

[cit20] Torrente-Murciano L., Hill A. K., Bell T. E. (2017). Catal. Today.

[cit21] Bell T. E., Menard H., Carballo J. M. G., Tooze R., Torrente-Murciano L. (2020). Int. J. Hydrogen Energy.

[cit22] Cahyanto H., Mazzei H. G., Tan A. F. J., Gu Q., Torrente-Murciano L., Yip A. C. K. (2026). ACS Sustainable Chem. Eng..

[cit23] Jana N. R., Chen Y., Peng X. (2004). Chem. Mater..

[cit24] Kura H., Takahashi M., Ogawa T. (2010). J. Phys. Chem. C.

[cit25] Costa N. J. S., Rossi L. M. (2012). Nanoscale.

[cit26] Pinho B., Torrente-Murciano L. (2020). React. Chem. Eng..

[cit27] Gao Y., Pinho B., Torrente-Murciano L. (2020). Curr. Opin. Chem. Eng..

[cit28] Pinho B., Torrente-Murciano L. (2021). Adv. Energy Mater..

[cit29] Hopper E. R., Wayman T. M. R., Asselin J. r. m., Pinho B., Boukouvala C., Torrente-Murciano L., Ringe E. (2021). J. Phys. Chem. C.

[cit30] Gao Y. H., Pinho B., Torrente-Murciano L. (2022). Chem. Eng. J..

[cit31] Wu K. J., Bohan G. M. D., Torrente-Murciano L. (2017). React. Chem. Eng..

[cit32] Pinho B., Williams L. M., Mahin J., Gao Y., Torrente-Murciano L. (2023). Chem. Eng. J..

[cit33] Mahin J., Torrente-Murciano L. (2020). Chem. Eng. J..

[cit34] Mahin J., Franck C. O., Fanslau L., Patra H. K., Mantle M. D., Fruk L., Torrente-Murciano L. (2021). React. Chem. Eng..

[cit35] Cargnello M., Doan-Nguyen V. V. T., Gordon T. R., Diaz R. E., Stach E. A., Gorte R. J., Fornasiero P., Murray C. B. (2013). Science.

[cit36] Zacharaki E., Beato P., Tiruvalam R. R., Andersson K. J., Fjellvåg H., Sjåstad A. O. (2017). Langmuir.

[cit37] Wu K. J., Torrente-Murciano L. (2018). React. Chem. Eng..

[cit38] Mehta J. P., Knappett B. R., Divitini G., Ringe E., Midgley P. A., Fairen-Jimenez D., Wheatley A. E. H. (2018). Part. Part. Syst. Charact..

[cit39] Dobbrow C., Schmidt A. M. (2012). Beilstein J. Nanotechnol..

[cit40] Ganley J. C., Thomas F. S., Seebauer E. G., Masel R. I. (2004). Catal. Lett..

[cit41] Tong W. P., Tao N. R., Wang Z. B., Lu J., Lu K. (2003). Science.

[cit42] Chen S., Chen X., Zhang H. (2017). Int. J. Hydrogen Energy.

[cit43] Zhang J., Xu H., Li W. (2005). Appl. Catal., A.

[cit44] Jedynak A., Kowalczyk Z., Szmigiel D., Raróg W., Zieliński J. (2002). Appl. Catal., A.

[cit45] Huang C., Yu Y., Tang X., Liu Z., Zhang J., Ye C., Ye Y., Zhang R. (2020). Appl. Surf. Sci..

[cit46] Ji J., Duan X., Qian G., Zhou X., Tong G., Yuan W. (2014). Int. J. Hydrogen Energy.

[cit47] Duan X., Qian G., Zhou X., Chen D., Yuan W. (2012). Chem. Eng. J..

[cit48] Xie P., Yao Y., Huang Z., Liu Z., Zhang J., Li T., Wang G., Shahbazian-Yassar R., Hu L., Wang C. (2019). Nat. Commun..

[cit49] Lu L., Zou S., Fang B. (2021). ACS Catal..

[cit50] Datta S., Torrente-Murciano L. (2018). Curr. Opin. Chem. Eng..

[cit51] Tang K., Liu W., Li J., Guo J., Zhang J., Wang S., Niu S., Yang Y. (2015). ACS Appl. Mater. Interfaces.

[cit52] Liu J., Zhao Z., Wang J., Xu C., Duan A., Jiang G., Yang Q. (2008). Appl. Catal., B.

[cit53] Reddy A. S., Chen C.-Y., Chen C.-C., Chien S.-H., Lin C.-J., Lin K.-H., Chen C.-L., Chang S.-C. (2010). J. Mol. Catal. A: Chem..

[cit54] Torrente-Murciano L., Gilbank A., Puertolas B., Garcia T., Solsona B., Chadwick D. (2013). Appl. Catal., B.

[cit55] Enger B. C., Holmen A. (2012). Catal. Rev.: Sci. Eng..

[cit56] Dry M. E. (2002). Catal. Today.

[cit57] van Deelen T. W., Nijhuis J. J., Krans N. A., Zečević J., de Jong K. P. (2018). ACS Catal..

[cit58] Niu F., Yan Z., Kusema B. T., Bahri M., Ersen O., Khodakov A. Y., Ordomsky V. V. (2020). ACS Catal..

[cit59] Yeung C. M. Y., Yu K. M. K., Fu Q. J., Thompsett D., Petch M. I., Tsang S. C. (2005). J. Am. Chem. Soc..

[cit60] Mei Z. J., Li Y., Fan M. H., Zhao L., Zhao J. (2015). Chem. Eng. J..

[cit61] Lucentini I., Casanovas A., Llorca J. (2019). Int. J. Hydrogen Energy.

[cit62] Cheng D., Gao Y., Yang H., Gong F., Yin G., Luo Y., Tian Y., Chen S., Du J., Yang J., Huang Z., Chen D. (2026). J. Catal..

