# Supplementary material for: Highly active earth-abundant ammonia cracking catalysts using long chain hydrocarbon stabilised colloidal nanoparticles
Source: Catal Sci Technol. 2026 Oct 26. Online ahead of print. doi: 10.1039/d6cy01170e (PMC13750304; doi:10.1039/d6cy01170e)
Supplement: Supplementary file 1 [file CY-OLF-D6CY01170E-s001.pdf]

Supplementary Information (SI) for Catalysis Science & Technology.
This journal is © The Royal Society of Chemistry

Highly active earth-abundant ammonia cracking catalysts using long chain hydrocarbon stabilised colloidal nanoparticles

*Julien Mahin, Joseph El-Kadi and Laura Torrente-Murciano**

Department of Chemical Engineering and Biotechnology, University of Cambridge, Philippa Fawcett Drive, CB3 0AS, Cambridge, UK

*Corresponding author: lt416@cam.ac.uk

Supplementary information

Table S1: Review of the state-of-the-art ammonia cracking catalysts using non-noble metals iron, cobalt, nickel or alloys in comparison to the activities reported in this study.

					Catalytic Performance Data					Surface area m2/g				
Catalyst Details	Metal content (w%)	NP size (nm)	T at ~1% conversion (°C)	Ea (kJ mol-1)	T(°C)	Conversion (%)	apparent rate molH2 molFe h-1	apparent rate molH2 gcat-1 h-1	GHSV (cm3NH3 gcat-1 h-1)	catalyst	support	Catalyst preparation	Ammonia %	Reference
Fe/CNT	2.8%	-	470	148.5	500	2.2%	79	0.039	30000	174	224	IWI of Fe(NO3)3 · 9H2O in acetone	100	Journal of Catalysis 224 (2004) 384–396
Fe/CMK-5	5.2%	6	-	-	500	26%	140	0.131	7500	1177	1783	IWI of Fe(NO3)3 · 9H2O in water	100	*J. Am. Chem. Soc.* 2010, 132, 40, 14152–14162
Fe/CNF,Mica	3.5%	85	400	93.5	500	44%	305	0.191	6500	55.6	-	IWI of Fe(NO3)3 · 9H2O in water	100	Applied Catalysis B: Environmental 101 (2011) 189–196
K-Fe/C	5.7%	12.5	-	166	470	-	345	0.352	85714	61	470	IWI of Fe(NO3)3 · 9H2O in water	20	Applied Catalysis A: General 237 (2002) 223–226
αFe_2O_3 @SiO_2	42%	50	430	-	500	18%	24	0.181	15000	382	-	Hydrothermal synthesis	100	Chem. Eur. J.2011,17, 598 – 605
Fe/ZSM-5	5%	6.9	475	-	500	12%	269	0.241	30000	-	363	IWI of Fe(NO3)3 · 9H2O in water	100	Molecular Catalysis 455 (2018) 14–22
Fe/CeO2	67%	21	210	21	500	93%	310	3.720	59760	25	-	Co-precipitation of Fe(NO3)3 · 9H2O in water	100	Materials Transactions, Vol. 43, No. 11 (2002) pp. 2763 to 2767
Fe/Al2O3 matrix	91%	10-20	-	127	500	50%	37	0.602	18000	77	-	Coprecipitation of Fe(NO3)3.9H2O and Al(NO 3)39H	100	J. Mater. Chem. A, 2015, 3, 17172–17180
Fe/CNT	5%	13	-	150	500	-	268	0.240	36000	-	-	impregnation	100	Nano Lett. 2008, 8, 9, 2738–2743
Fe/La2O3	15%	10-50	400	-	500	0.47	211	0.566	18000	59	76	coprecipita-tion-hydrothermal metho	100	J. Rare Earths 2017, 35 (1), 15–23, DOI: 10.1016/S1002-0721(16)60167-9
Fe3O4	100%	100	400	-	500	0.5	45	0.803	24000	20	-	precipitation	100	Sci. Bull. 2016, 61 (3), 220–226
Fe@GC	1.3%	12.7	400	-	500	0.11	191	0.044	6000	447	-	hydrothermal	100	Mol. Catal. 2017, 442, 147–153
Fe/La-MgO	20%	25	400	197.3	500	0.43	177	0.633	22000	101	125	deposition precipitation	100	J. Energy Chem. 2019, 38, 41–49
Fe@CNT	2.8%	10-50	-	-	500	0.08	53	0.027	5000	-	-	CVD	100	(2007) Chem Commun 19:1916–1918
**Fe/CeO2**	**0.20%**	**4.4**	**474**	**156**	**500**	**3%**	**280**	**0.010**	**6000**	**-**	**-**	**CIWI**	**29**	**This work**
**Fe/CeO2 (calcined)**	**0.20%**	**4.4**	**478**	**131**	**500**	**6%**	**673**	**0.024**	**6000**	**-**	**-**	**CIWI**	**29**	**This work**
**Fe/CeO2**	**2.39%**	**10.8**	**415**	**190**	**500**	**28%**	**263**	**0.112**	**6000**	**-**	**-**	**CIWI**	**29**	**This work**
**Fe/CeO2 (calcined)**	**2.39%**	**10.8**	**430**	**169**	**500**	**33%**	**310**	**0.133**	**6000**	**-**	**-**	**CIWI**	**29**	**This work**
Co in Al2O3 matrix	95%	10-20	400	123	500	72%	54	0.867	18000	74	-	Co-precipitation of Co and Al nitrates	100	Journal of Materials Chemistry A, 2015. 3(33): p. 17172-17180.
Co/MgO-La2O3	5%	2.5	300	67	500	60%	284	0.241	6000	30.7	42	Co-precipitation of Co, Mg and La nitrates	100	Journal of Molecular Catalysis A: Chemical, 2016. 414: p. 130-139.
Co/MgO-La2O3	5%	8.8	300	-	500	53%	251	0.213	6000	1	30*	Co-precipitation of Co, Mg and La nitrates in O2	100	International Journal of Hydrogen Energy, 2017. 42(38): p. 24213-24221.
Co-SiO2 composite	42%	5-10	400	155	500	0%	150	0.001	22000	-	277*	Impregnation of Co(NO3)2 (aq) into silicate - activated carbon (AC) support and calcined at 500°C	100	Chempluschem, 2017. 82(3): p. 368-375
Co3O4-Al2O3	69%	30	300	148	500	44%	45	0.530	18000	-	57*	Aerosol spray of mix of copolymer and Co and Al nitı	100	Science China Chemistry, 2018. 61(11): p. 1389-1398
Co/Ax-21	7%	-	330	89	500	59%	173	0.205	5200	-	2257	Incipient wetness impregnation Co(NO3)2 (aq)	-	Catalysis Today, 2017. 286: p. 131-140.
Co/CNT	7%	-	355	94	500	27%	79	0.094	5200	-	253	Incipient wetness impregnation Co(NO3)2 (aq)	-	Catalysis Today, 2017. 286: p. 131-140.
Co/γ-Al2O3	7.6%	7.7	350	105	500	53%	165	0.213	6000	-	-	Precipitation with Co(NO3)2 (aq) and sodium carbonate buffer.	42	International Journal of Hydrogen Energy, 2020. 45(51): p. 27210-27220
Co/TiO2 nanotubes	3.5%	15	400	70	500	13%	88	0.052	6000	49	-	NaOH deposition-precipitation with Co(NO3)2 (aq)	42	International Journal of Hydrogen Energy, 2019. 44(57): p. 30062-30074
Co/SiC	5.2%	10	300	104	450	92%	218	0.192	3000	-	25	Impregnation of Co(NO3)2 (aq)	5	Catalysis Today, 2022. 390-391: p. 34-47
Co/SiC with K promoter	4.1%	10	300	98	450	98%	245	0.170	3000	-	-	Co-impregnation with K nitrate	5	Catalysis Today, 2022. 390-391: p. 34-47
Co/BaAl12O19	35%	-	350	70	500	87%	294	1.747	30000	-	6.89	Wet Impregnation of Co(NO3)2 (aq)	100	Catalysis Today, 2022. 402: p. 45-51
Co/CeO2	42%	5-30	300	90	500	100%	75	0.529	9000	3.95	-	Co-precipitation	100	Catal. Sci. Technol., 2021, 11, 3014
Ba/Co/CeO2	41%	5-30	300	81	500	100%	79	0.552	9000	4.45	-	Co-precipitation	100	Catal. Sci. Technol., 2021, 11, 3014
Co/BaCeO3 (perovskite)	5%	-	300	70	500	56%	265	0.225	6000		14.2	Wet Impregation of Co(NO3)2 (aq)	100	Arabian Journal for Science and Engineering, 2023, 48: p. 8667–8677
Co/3D-meso CeO2	5%	5.2	300	65	500	62%	297	0.252	6000	-	90	Incipient wetness impregnation Co(NO3)2 (aq)	100	Applied Surface Science, 2020, 532, 147335
Co/nitrogen doped carbon	23%	4-10	300	75	500	80%	410	1.620	30000			Mixing Co(NO3)2 (aq) with 2-methylimidazole in methanol, then dry and calcine	100	International Journal of Hydrogen Energy, 47(2022) 12882-12892
Co/BaNH	5%	5.3	250	46	500	19%	1473	**1.200**	36000	-	11.8*	Ball-milling anhydrous CoCl2 with Ba(NH2)2	100	Journal of Energy Chemistry, 2020, 46: p. 16-21
**Co/CeO2**	**1.1%**	**10.7**	**350**	**90**	**500**	**35%**	**781**	**0.141**	**6000**	**-**	**-**	**CIWI**	**29**	**This work**
**Co/CeO2 (calcined)**	**1.1%**	**10.7**	**350**	**93**	**500**	**45%**	**1005**	**0.181**	**6000**	**-**	**-**	**CIWI**	**29**	**This work**
**Co/CeO2**	**0.7%**	**2.4**	**370**	**93**	**500**	**21%**	**696**	**0.083**	**6000**	**-**	**-**	**CIWI**	**29**	**This work**
**Co/CeO2 (calcined)**	**0.7%**	**2.4**	**380**	**90**	**500**	**17%**	**571**	**0.068**	**6000**	**-**	**-**	**CIWI**	**29**	**This work**
**Co/Al2O3**	**1.59%**	**10.7**	**435**	**99**	**500**	**4%**	**63**	**0.017**	**6000**	**-**	**-**	**CIWI**	**29**	**This work**
**Co/CNT**	**2.04%**	**10.7**	**470**	**105**	**500**	**2%**	**18**	**0.006**	**6000**	**-**	**-**	**CIWI**	**29**	**This work**

Ni/CNT	2.9%	-	400	90.3	500	9%	352	0.174	30000	170	224	IWI of Ni(NO3)26H2O in acetone	100	Journal of Catalysis 224 (2004) 384–396
Ni/zeolite13X	14.8%	21.9	425	57	500	20%	159	0.402	30000	563	919	IWI of Ni(NO3)26H2O	100	Sustainable Energy Fuels, 2024, Advance Article
Ni/Al2O3(La)	37.9%	3.9	380	-	500	64%	199	1.283	30000	-	143	Coprecipitation of Ni(NO3)26H2O and Al(NO 3)39H	100	Applied Catalysis A: General 290 (2005) 87–96
Ni/Al_2O_3(Ce)	38.3%	6.5	-	-	500	72%	221	1.444	30000	139	-	Coprecipitation of Ni(NO3)26H2O and Al(NO 3)39H	15	Applied Catalysis B: Environmental 80 (2008) 98–105
Ni/SBA-15	23.4%	4.3	-	-	500	57%	287	1.144	30000	365	838.5	Deposition–precipitation from Ni(NO3)26H2O	100	Applied Catalysis A: General 337 (2008) 138–147
Ni/Al2O3/monolith	15%	6	400	153	500	21%	192	0.492	35,000	258	-	Adsorption of Ni(NO3)26H2O	100	Journal of Catalysis 275 (2010) 228–235
Ni@SiO2	83%	5-50	-	-	500	35%	50	0.703	30000	112	-	Precipitation of Ni(NO3)26H2O and encapsulation	100	Catalysis Today 158 (2010) 401–408
Ni/Al2O3 matrix	91%	10-20	-	123	500	59%	46	0.711	18000	121	-	Coprecipitation of Ni(NO3)26H2O and Al(NO 3)39H	100	J. Mater. Chem. A, 2015, 3,17172
Ni/TiO2	40%	-	400		500	12%	7	0.048	6000	-	12	IWI of Ni(NO3)26H2O in acetone	100	RSC Adv., 2018, 8, 32102
Ni/CaTiO3	40%	-	400		500	15%	9	0.060	6000	-	6.5	IWI of Ni(NO3)26H2O in acetone	100	RSC Adv., 2018, 8, 32103
Ni/SrTiO3	40%	-	375		500	42%	25	0.169	6000	-	5.5	IWI of Ni(NO3)26H2O in acetone	100	RSC Adv., 2018, 8, 32104
Ni/BaTiO3	40%	-	375		500	33%	19	0.133	6000	-	4.9	IWI of Ni(NO3)26H2O in acetone	100	RSC Adv., 2018, 8, 32105
Ni/ZrO2	40%	37.4	400		500	11%	6	0.044	6000	-	15	IWI of Ni(NO3)26H2O in acetone	100	RSC Adv., 2018, 8, 32106
Ni/CaZrO3	40%	48.4	375		500	22%	13	0.088	6000	-	6	IWI of Ni(NO3)26H2O in acetone	100	RSC Adv., 2018, 8, 32107
Ni/SrZrO3	40%	57.6	350		500	61%	36	0.245	6000	-	6.1	IWI of Ni(NO3)26H2O in acetone	100	RSC Adv., 2018, 8, 32108
Ni/BaZrO3	40%	99.2	350		500	66%	39	0.265	6000	-	7.4	IWI of Ni(NO3)26H2O in acetone	100	RSC Adv., 2018, 8, 32109
Ni/SiO2	10%	-	400		500	0.31	73	0.124	6000		280	100	(NO3)26H2O ir	Appl. Catal. A: General 2012, 443–444, 119–124
Ni/Al1Ce0.05Ox	10%	3.5;14	350	74.72	500	0.76	179	0.305	6000	274	-	100	tion double hy	Appl. Catal. B: Environmental 2022, 307, 121167
Ni/ZSM-5	5%	6.9	400	88.1	500	0.41	966	0.823	30000	385	385	100	solid state ion	Appl. Catal. A: General 2018, 562, 49–57
Ni/MWCNT	5%	7.4	400	-	500	0.57	269	0.229	6000	-	169.3	100	(NO3)26H2O ir	Int. J. Hydrogen Energy 2014, 39 (1), 277–287
Ni/Ac	5%	6.1	400	-	500	0.25	118	0.100	6000	-	2954	100	(NO3)26H2O ir	Int. J. Hydrogen Energy 2014, 39 (1), 277–287
Ni/rGO	10%	5-10	400	65.34	500	0.13	153	0.261	30000	-	-	100	Precipitation	ournal of Industrial and Engineering Chemistry 2015, 32, 373–379
Ni/graphene aerogel	11%	13.6	-	94.8	500	-	171	0.324	30000	581	-	100	impregnation	Energy Technol. 2022, 10, 2100794
Ni/mica	5%	11.3	375	-	500	0.22	518	0.442	30000	73	83	100	npregnation m	Int. J. Hydrogen Energy 2018, 43 (20), 9663–9676
Ni/red mud	15%	14	400	70.3	500	0.15	118	0.301	30000	225	219	100	cipitation metl	Catal. Sci. Technol. 2014, 4 (2), 361–368
Ni/clay	30%	14	400	83.8	500	0.12	47	0.241	30000	228.5	77.2	100	cipitation metl	Int. J. Hydrogen Energy 2016, 41 (46), 21157–21165
Ni/AlMg-LDH	40%	6.5	400	-	500	0.42	123	0.843	30000	-	-	100	hydrothermal	Appl. Catal. B: Environmental 2017, 201, 451–460
Ni/Ce0.8Zr0.2O2	4%	-	350	54	500	0.6	236	0.145	3600	221	-	100	impregnation	Int. J. Hydrogen Energy 2012, 37 (21), 15901–15907
Ni/CaNH	10%	16.2	350	88	500	0.7	412	0.703	15000	-	18	100	reduction	ACS Catal. 2021, 11 (17), 11005–11015
**Ni/CeO2**	**0.095%**	**3**	**416**	**103**	**500**	**4%**	**1060**	**0.017**	**6000**	**-**	**-**	**CIWI**	**29**	**This work**
**Ni/CeO2 (calcined)**	**0.095%**	**3**	**420**	**123**	**500**	**6%**	**1496**	**0.024**	**6000**	**-**	**-**	**CIWI**	**29**	**This work**
**Ni/CeO2**	**0.31%**	**2.4**	**370**	**95**	**500**	**40%**	**3019**	**0.161**	**6000**	**-**	**-**	**CIWI**	**29**	**This work**
**Ni/CeO2 (calcined)**	**0.31%**	**2.4**	**355**	**98**	**500**	**33%**	**2491**	**0.133**	**6000**	**-**	**-**	**CIWI**	**29**	**This work**
**Ni/Al2O3**	**0.18%**	**2.4**	**385**	**113**	**500**	**11%**	**1427**	**0.044**	**6000**	**-**	**-**	**CIWI**	**29**	**This work**
CoMo/γ-Al2O3	5%	1.8	360	-	500	54%	**2030**	1.311	36000	179.5	154.7	IWI with Co(en)$_3$MoO$_4$	100	Int. J, Hydr. Energy 3 9 (2 0 1 4) 1 2 4 9 0 e1 2 4 9 8
CoMoFeNiCu/CNF	7.80%	20	300	65	500	84%	**1728**	2.024	36000	157	-	carbothermal shock	5	*Nat Commun* **10**, 4011 (2019)
Co3Mo3N (Cs)	97%	30–80	320	65.1	500	95%	**30**	0.380	6000	8.2	-	Calcination	100	Applied Catalysis B: Environmental 218 (2017) 1–8
FeMo/Al2O3 (La)	5%	12	430	-	500	8.7%	**407**	0.268	46000	92	102	IWI with Fe(acac)3 and MoO2(acac)2 in acetone	100	*Applied Catalysis B: Environmental 125 (2012) 409–417*
Ni_2Mo_3N	97%	-	380	-	500	29%	**35**	0.419	21,600	6.1	-	acetate evaporation	100	Int. J, Hydr. Energy 4 1 (2 0 1 6) 3 8 5 4 e3 8 6 0
Co-Ni/SiO2	5% Co<br>5% Ni	<10	350	54	500	46%	**543**	0.924	30000	-	300	Wet co-impregnation with Ni and Co nitrate	100	International Journal of Hydrogen Energy, 2020, 45, p. 15263-15269
$CoMo_2N$	93%	14	350	92.8	500	39%	**16**	0.157	6000	93	-	Co-precipitation and nitridation	100	Catal. Sci. Technol., 2016,6, 1496-1506
CoMo/MCM-41	5%	2–4	400	-	500	52%	**2126**	1.253	36000	668.2	841	incipient wetness impregnation with an aqueous solution of precursors (Co(NO 3)26H2O and(NH4)6 Mo7O 244H2O	100	Chemical Engineering Journal 207–208 (2012) 103–108
CoNi/CeZrY	9%	-	325	41.1	500	60%	**158**	0.241	6000	-	-	Co-impregnation of Co and Ni nitrates	100	Applied Surface Science 478 (2019) 708–716

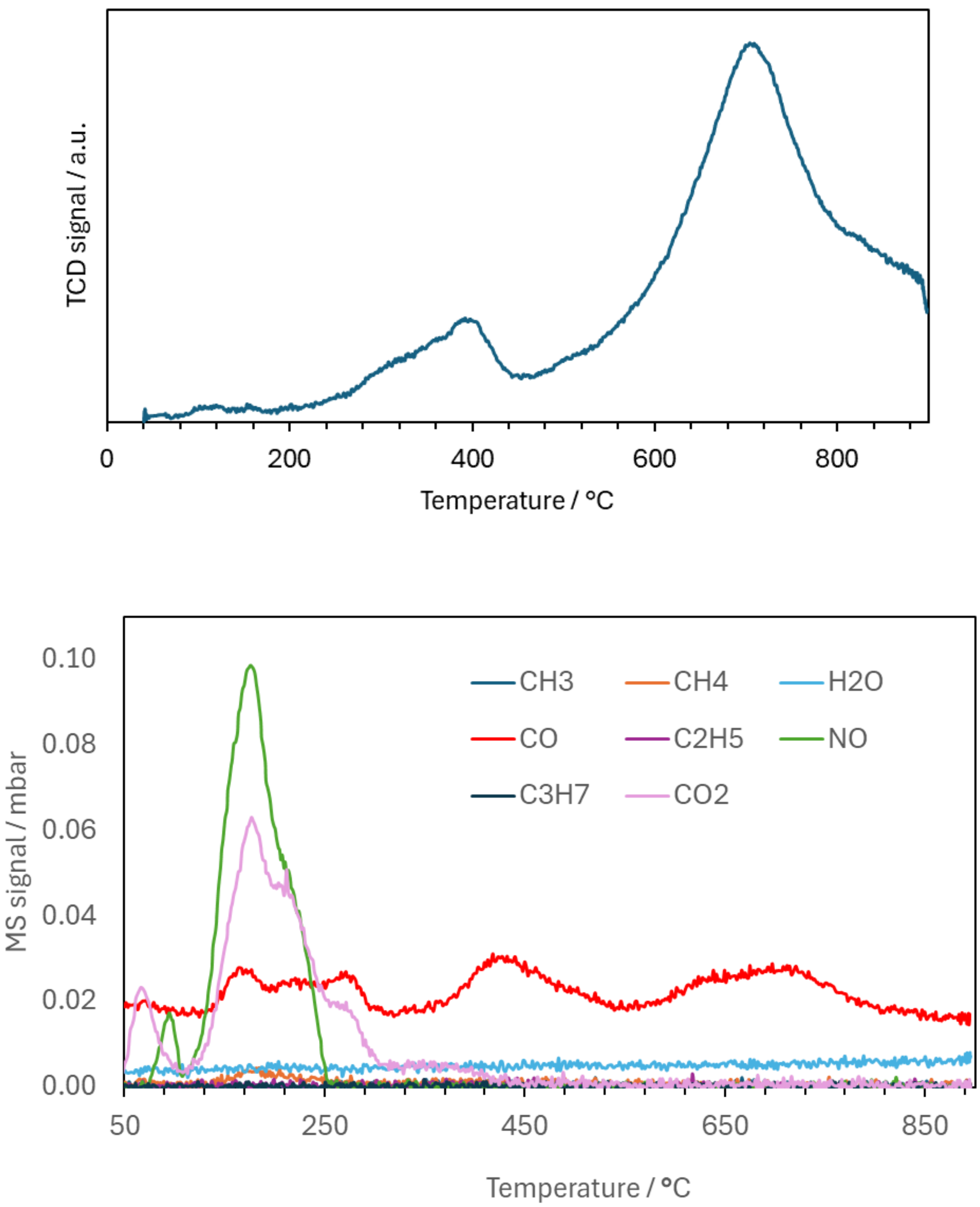

Figure S1: (top) H_2-TPR profile of pure CeO_2 (Sigma-Aldrich). The first peak corresponds to the reduction of surface Ce(IV) to Ce(III) while the second peak corresponds to bulk Ce(IV) -> Ce(III) reduction (top) and (bottom) MS spectra of the gases generated (bottom).

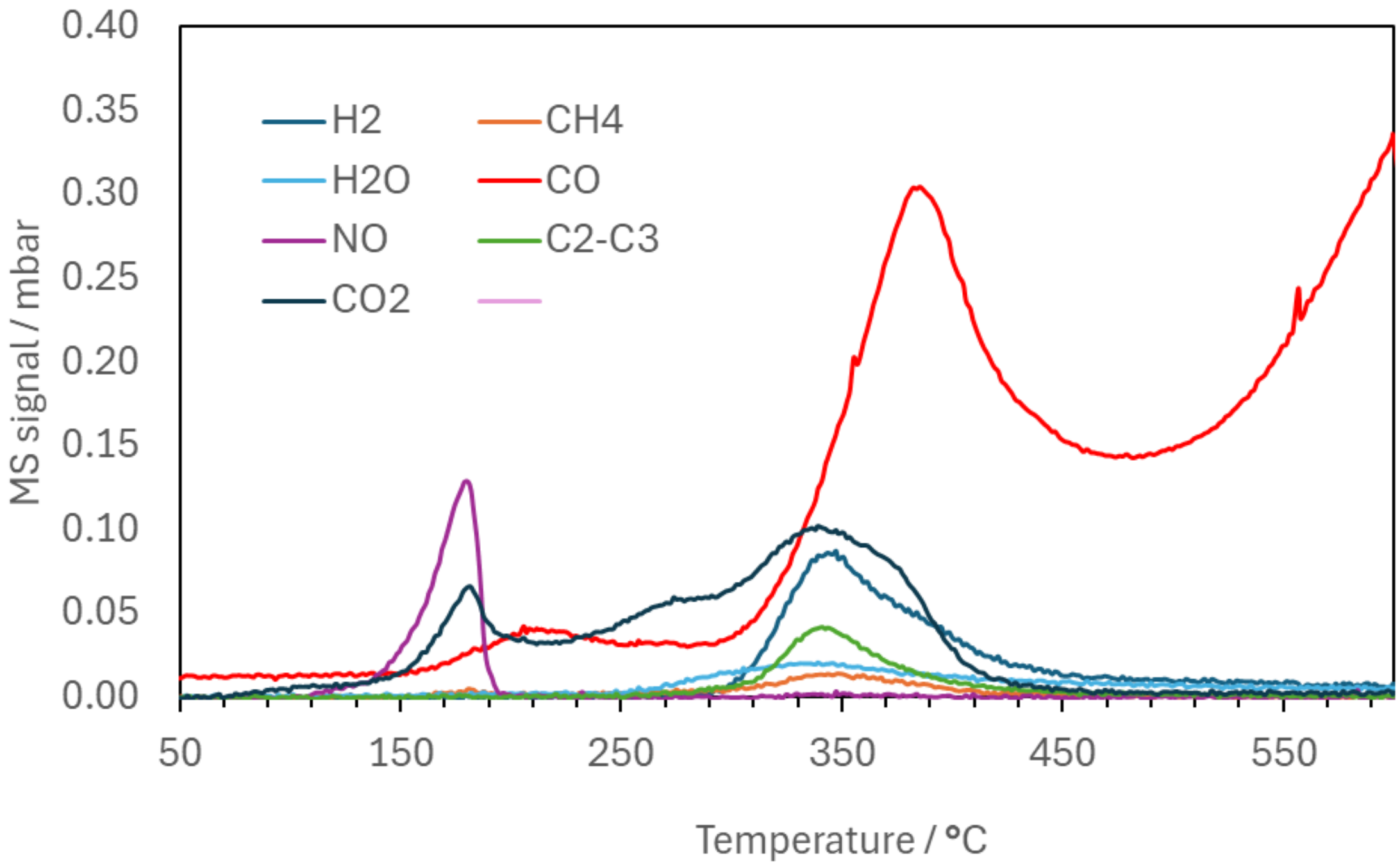

Figure S2: MS spectra of the gases generated by CoNP/CeO_2 temperature programmed calcination in argon, showing a small CH_4 peak and large hydrogen generation peak.

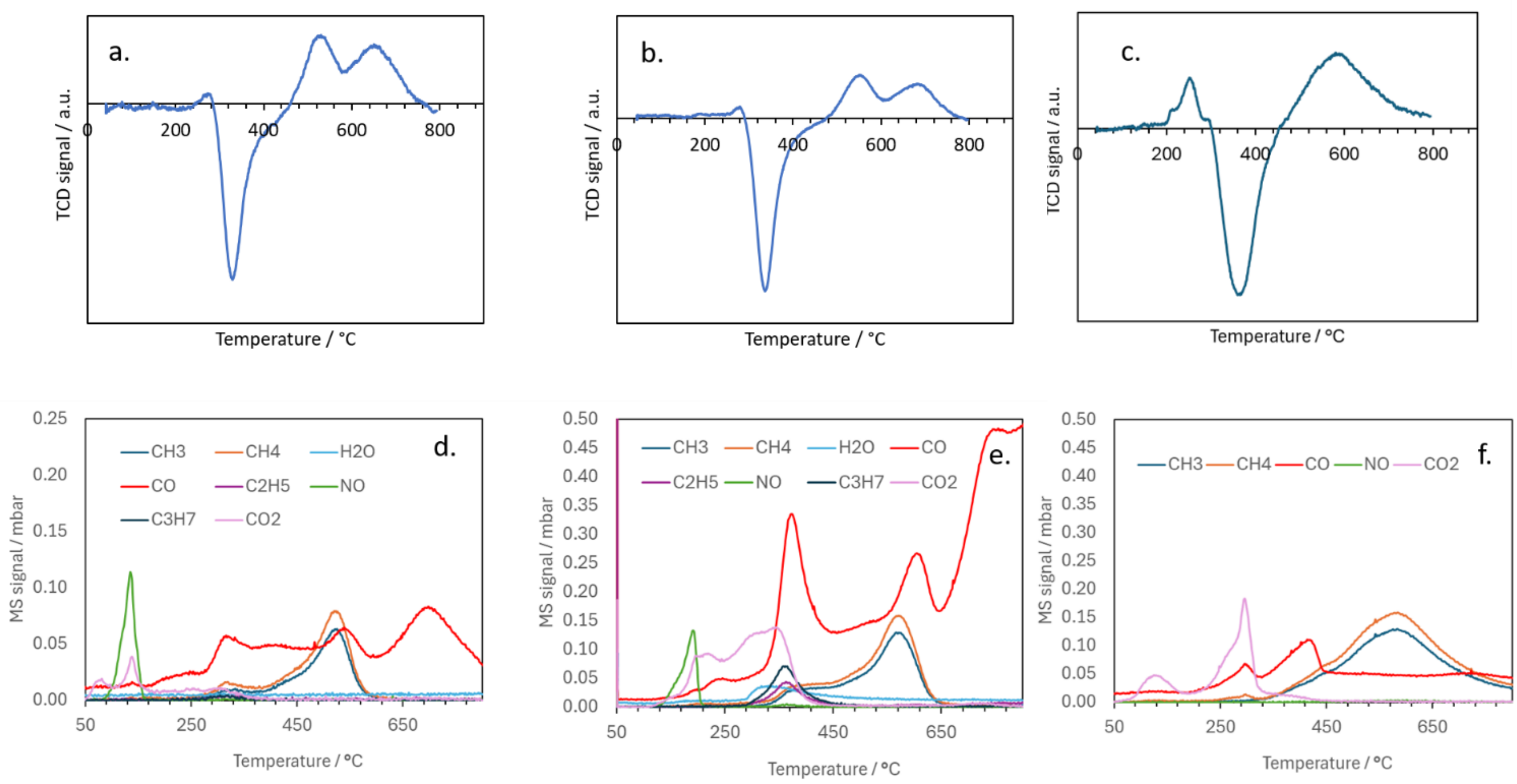

Figure S3: H_2-TPR profiles and corresponding MS spectra for cobalt nanoparticles (10.7 nm) deposited on CeO_2 (a, d), Al_2O_3 (b, e) and MWCNT (c, f).

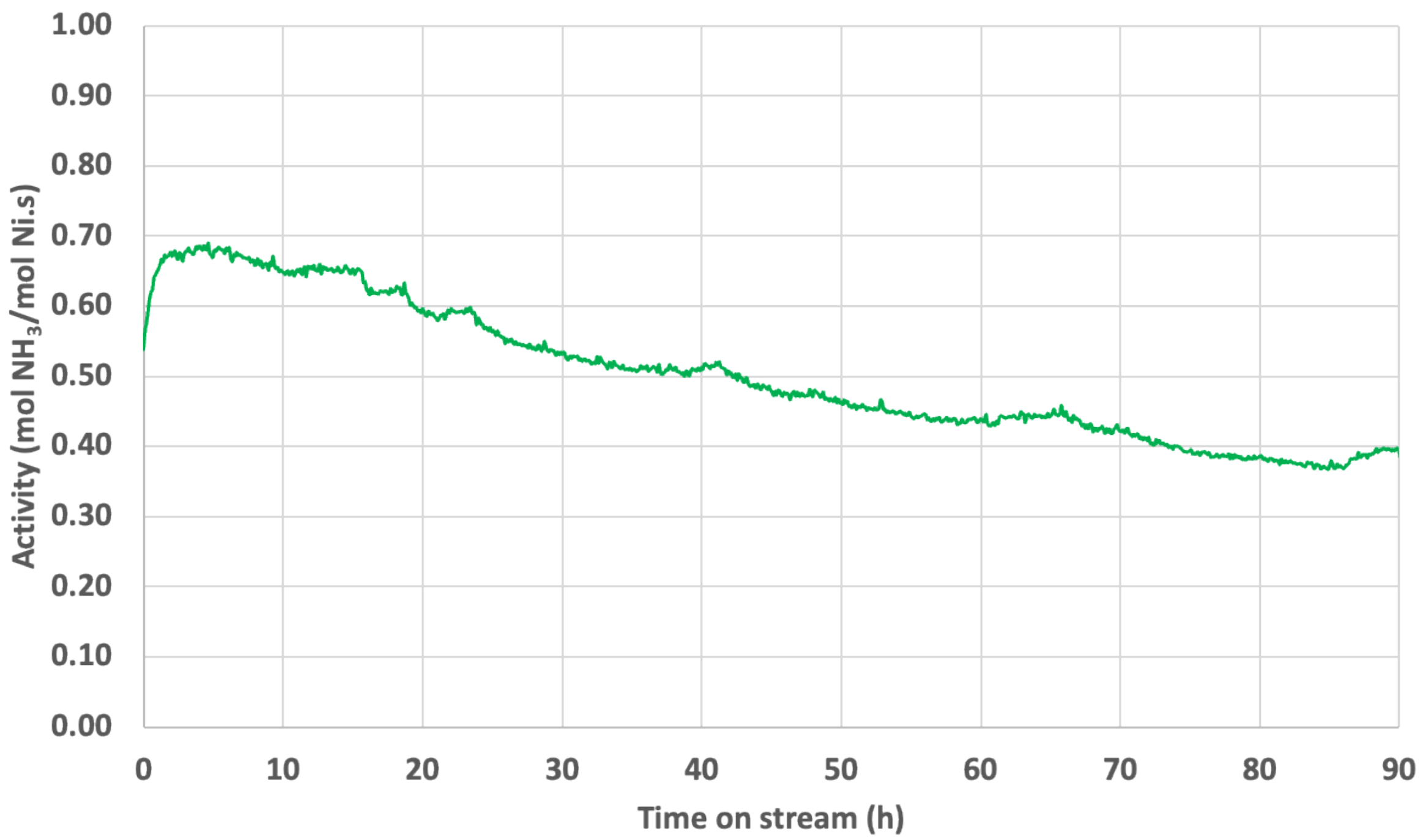

Figure S4: Evolution of the activity of 2.4 ± 0.3 nm nickel nanoparticle catalyst on CeO_2 support over 90h at 500°C. The catalytic test conditions were the same as those described for the other ammonia decomposition tests (GHSV of 6000 $mLNH_3 \cdot g_{cat}^{-1}$ h^{-1}).

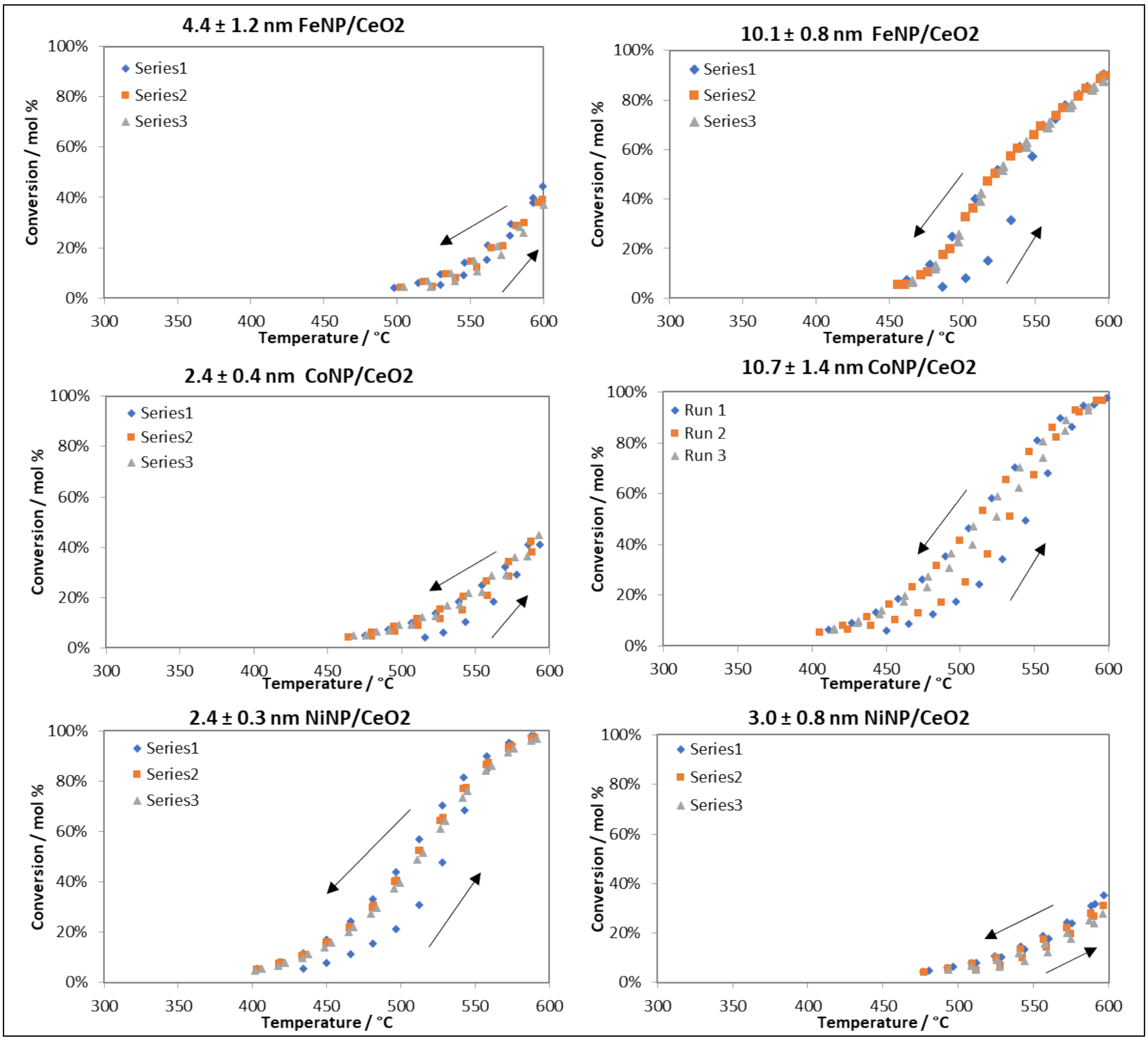

Figure S5: Full catalytic activity data for NH_3 decomposition of different metal catalysts on CeO_2, with ligands initially present on the nanoparticle surface. All the catalysts were reduced for 30 min in pure flowing H_2 at 400°C. No calcination treatment was performed on these samples. Series 1, 2 and 3 refer to the temperature cycles where temperature is initially increased to 600°C and then decreased to 200°C.

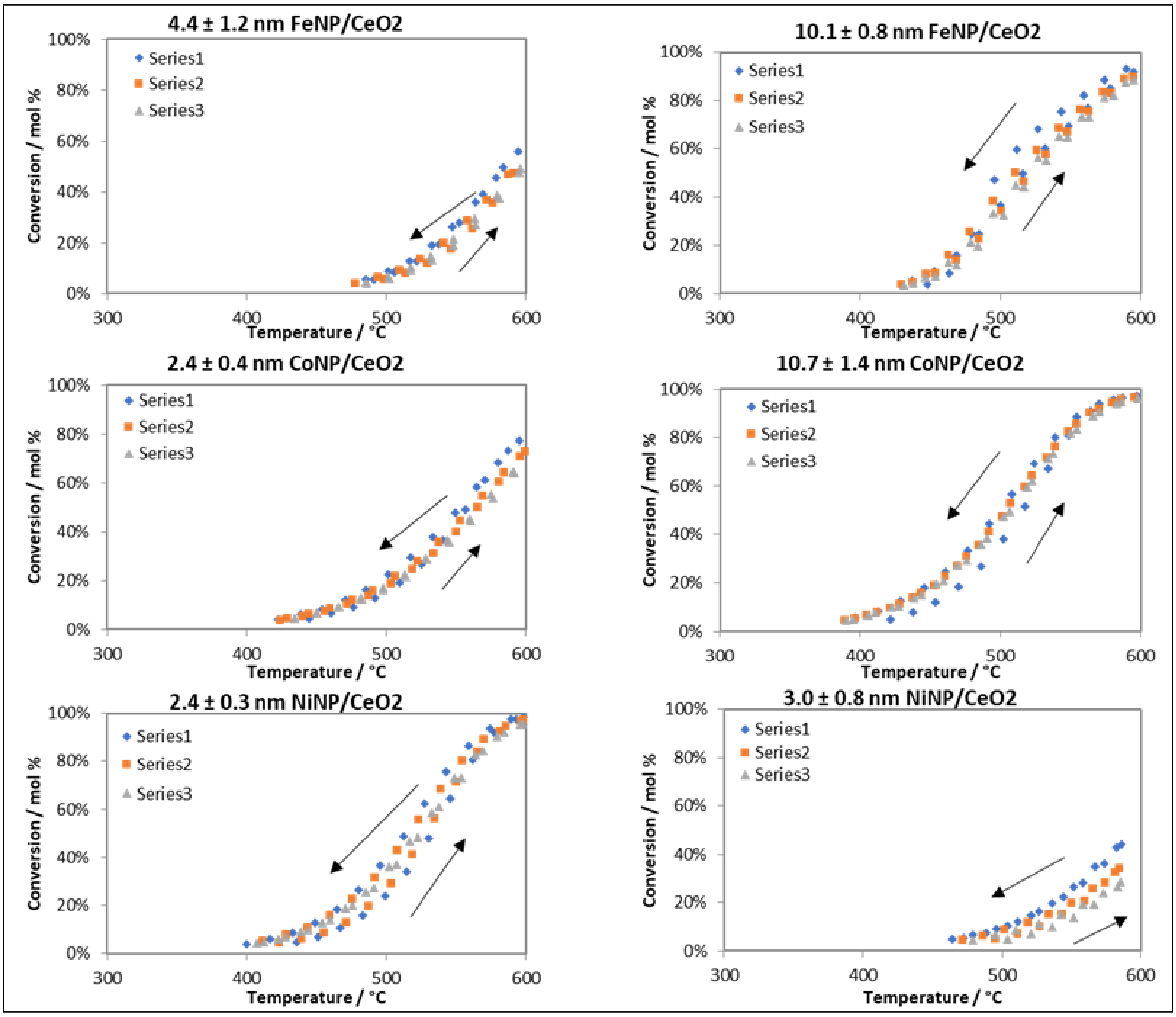

Figure S6: Full catalytic activity data for NH_3 decomposition of different metal catalysts on CeO_2, with ligands initially removed by calcination in 5% O_2 at 300°C for 2h. All the catalysts were reduced for 30 min in pure flowing H_2 at 400°C. Series 1, 2 and 3 refer to the temperature cycles where temperature is initially increased to 600°C and then decreased to 200°C.

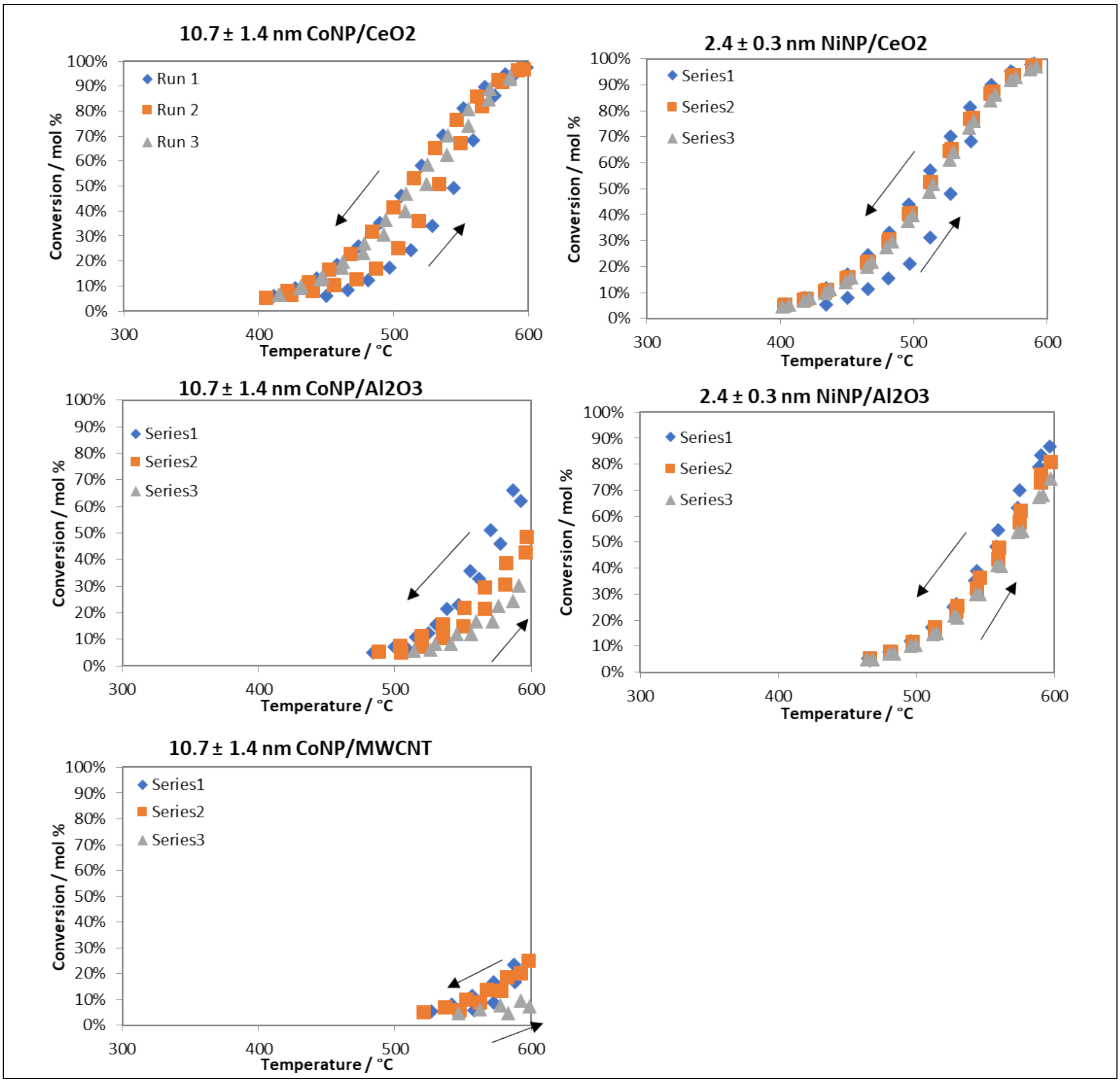

Figure S7: Full catalytic activity data for NH_3 decomposition of cobalt nanoparticle catalysts on CeO_2, Al_2O_3 and MWCNT supports. All the catalysts were reduced in situ at 400°C in H_2 for 30 min prior to the catalytic tests. Series 1, 2 and 3 refer to the temperature cycles where temperature is initially increased to 600°C and then decreased to 200°C.
